# COVID-19 vaccination coverage and the effect on regional disparities in morbidity and mortality among older people in Sweden, 2021–2023

**DOI:** 10.1177/14034948261420643

**Published:** 2026-02-23

**Authors:** Anton Nilsson, Dominik Dietler, Carl Bonander, Malin Inghammar, Jonas Björk

**Affiliations:** 1 Epidemiology, Population Studies and Infrastructures (EPI@LUND), Division of Occupational and Environmental Medicine, Epidemiology, Lund University, Lund, Sweden; 2 Register-based Epidemiology, Department of Translational Medicine, Lund University, Malmö, Sweden; 3 School of Public Health and Community Medicine, Institute of Medicine, University of Gothenburg, Gothenburg, Sweden; 4 Infection Medicine, Department of Clinical Sciences Lund, Lund University, Lund, Sweden; 5 Department of Infectious Diseases, Skåne University Hospital, Lund, Sweden; 6 Clinical Studies Sweden, Forum South, Skåne University Hospital, Lund, Sweden

**Keywords:** COVID-19, mortality, hospitalizations, vaccination, regional disparities

## Abstract

**Aims:**

The COVID-19 pandemic had substantial impacts on mortality and morbidity, even after the rollout of vaccinations. These impacts however, varied considerably across regions. We examined regional disparities in COVID-19 mortality and hospitalizations among older people in Sweden from January 2021 to August 2023 and assessed the role of vaccination coverage in shaping these disparities.

**Methods:**

Using full-population data on people in Sweden born in 1955 or earlier, we created a nested case-control study. Conditional logistic regressions with county fixed effects were estimated across three periods of vaccination rollout (Doses 1–2, 3–4, and 5–6). County fixed effects were compared across models with and without vaccination adjustment. Further, the benefits of vaccination coverage corresponding to the most successful regions were evaluated with potential impact fractions (PIFs).

**Results:**

Across counties, the final shares of vaccinated individuals ranged from 94% to 98% for Doses 1 and 2, 90% to 96% for Dose 3, 80% to 91% for Dose 4, 65% to 83% for Dose 5, and 13% to 47% for Dose 6. In models without vaccination adjustments, COVID-19 mortality ranged from −56% to +75% relative to an average county, and hospitalizations from −45% to +51%. Vaccination adjustment had little impact on the disparities. Overall PIFs were 8% to 11% for mortality and 7% to 8% for hospitalizations, with greater PIFs in urban counties.

**Conclusions:**

**While higher vaccination coverage, especially in urban areas, could have prevented COVID-19 deaths and hospitalizations among older adults in Sweden, the vaccinations were not a major driver of regional disparities in these outcomes.**

## Introduction

The COVID-19 pandemic has had substantial impacts on mortality and morbidity around the world, even after the implementation of vaccinations [1, 2]. The impact of the pandemic has, however, varied considerably across regions [3–10], raising concerns and prompting calls for the implementation of policies to counter these inequalities [11–13]. To devise effective policies, the factors responsible for the diverse pandemic outcomes across areas need to be identified.

Several studies have found that COVID-19 infections and deaths covary with vaccination coverage across countries or regions [14–17], with lower COVID-19 mortality often being observed in countries or areas with higher vaccination coverage. One major limitation of these studies is that analyses relying on aggregate data can be subject to ecological biases [18]. To avoid this problem, data at the individual level are needed. However, individual-level data with information on vaccination, death, and health outcomes, together with confounders such as comorbidities and sociodemographic characteristics, are in general not available to researchers, except in a few settings, such as in the Nordic countries.

In this study, we examined regional disparities in deaths and hospitalizations due to COVID-19 during the post-vaccination period of the COVID-19 pandemic among older people in Sweden, the largest Nordic country. We studied disparities across Sweden’s 21 administrative regions (counties) and assessed to what extent differences in the uptake of the COVID-19 vaccination may explain regional disparities in the outcomes. In Sweden, county-level health inequalities are of particular interest, as healthcare is organized at this scale, and there are known regional differences in the accessibility and quality of care [19]. Sweden’s pandemic response, including its age-based vaccination recommendations and the allocation of vaccines to the counties, was centrally coordinated by the Public Health Agency of Sweden. However, regions differed in how vaccinations were implemented, including where and when vaccinations were offered, how residents were informed, booking systems, and the pace of the rollout. National coordination and central funding of the pandemic response were strongest during the early phases of the pandemic and gradually decreased over time [20]. Regional disparities in the uptake of the COVID-19 booster vaccine doses across Swedish counties have been highlighted previously, and have been attributed, at least in part, to differences in information about the vaccinations provided to the residents as well as to socioeconomic factors, country of birth, and local neighborhood factors [20, 21].

We assessed the role of vaccinations in shaping regional disparities in the outcomes by contrasting estimates of county fixed effects across models with and without adjustment for vaccination uptake. In addition, we calculated potential impact fractions (PIFs) to assess the hypothetical benefit of a scenario where vaccination coverage in each age- and sex-stratum matched that of the most successful county for that stratum.

## Data and methods

### Data sources

Our study was based on the SHIELD (Surveillance of Health Threats from Infectious Diseases among Older Persons) cohort, a linkage of individual-level data from several full-population administrative registers. These data comprised all inhabitants in Sweden born in 1955 or earlier who were alive on January 1, 2021—essentially the time when COVID-19 vaccinations began. Data were available through August 2023.

Data included birth year, birth country (Sweden or abroad), sex (male or female), civil status (married/registered partner or other), county of residence (21 counties), and 2020 aggregate educational attainment at the RegSO level (a division of Sweden into 3363 areas), provided by Statistics Sweden. Moreover, we used data on hospitalizations, outpatient specialist visits, deaths, and nursing home or home care status by month, from the National Board of Health and Welfare, and data on COVID-19 vaccinations from the Public Health Agency of Sweden. Causes of deaths and healthcare visits were expressed with ICD-10 codes (three and four characters, respectively).

### Variable constructions

Outcomes included COVID-19 deaths and hospitalizations. COVID-19 hospitalizations were defined as hospitalizations with ICD-10 codes U071, U072, or U109 as the main diagnosis code. COVID-19 deaths were defined as deaths where the ICD code for the main cause of death began with U07 or U10.

Analyses were adjusted for comorbidities that are established risk factors for severe COVID-19 [22, 23]: cardiovascular diseases (ICD-10 codes I10–I15, I20–I25, I42, I43, I50, I6, and J81), diabetes and obesity (E10, E11, and E66), kidney and liver diseases (K70, K743–KK746, K754, K760, N185, and N189), respiratory diseases (A15–A19, E84, I26, I27, J42–J45, J47, J84, J96, J98.2, and J98.3), neurological conditions including dementia (F00–F03, G00–G99), cancer or immunosuppressed state (C00–C99, D800, D801, D805, D81–D83), and other conditions and diseases (B20–B24, D56, D57, F1–F3, Q90, Z94, and T86), based on main and secondary diagnoses from hospitalizations and outpatient visits.

Vaccination status was measured by time since last dose (7–90 days, 91–179 days, 180–269 days, 270–365 days, or more than 365 days), and the number of doses received (0, 1, 2, 3, 4, 5, or 6 or more). Due to the immune response delay, vaccinations taking place in the past 7 days were not included when measuring vaccination status.

### Statistical methods

#### Construction of case-control data

To make regression estimation computationally feasible, we created a nested case-control dataset within the study cohort, as follows. For each individual experiencing an outcome (COVID-19 death or hospitalization), we drew 10 controls of the same sex and year of birth among the alive individuals who had not yet experienced the outcome on the event day. Individuals could appear as controls in the matching sets of several cases, and could also later appear as cases. This sampling design, known as *density sampling*, allowed us to estimate effects with conditional logistic regression (conditional on the matching units), with the estimated odds ratios (ORs) corresponding to incidence rate or mortality ratios [24].

#### Regressions and fixed effects comparisons

We employed two separate sets of conditional logistic regression models. Regression models referred to as “Model 1” were adjusted for county fixed effects, demographics, living arrangement (receiving home services, and living in a nursing home), and disease history (during the 5 years prior to the outcome). In further models, referred to as “Model 2,” we additionally adjusted for vaccination status. Models were estimated in three periods: Period 1, covering the rollouts of Doses 1 and 2 (January 1, 2021–September 27, 2021), Period 2, covering Doses 3 and 4 (September 28, 2021–August 31, 2022), and Period 3, covering Doses 5 and 6 (September 1, 2022–August 31, 2023).

In Model 1, the county-specific ORs quantify the relative incidence of outcomes between individuals in a comparison and reference county, while holding demographics, living arrangement, and disease history fixed. These ORs reflect all county-specific factors that influence the outcome but are not captured by other covariates. These factors may include local SARS-CoV-2 transmission dynamics, population immunity due to previous infections, healthcare organization, availability of information, and vaccination coverage. The interpretation of the ORs in Model 2 is similar, except that vaccination coverage is additionally held fixed, meaning that we compare individuals similar not only with respect to demographics, living arrangement, and disease history, but also with respect to vaccination status. An assessment of how much the ORs differ across Models 1 and 2 hence allows us to gauge the importance of vaccinations in shaping differences in the outcomes across counties. The delta method was used to test whether estimates from the two models differed.

To avoid relying on an arbitrary reference county, county effects were reported using normalized ORs, defined as the corresponding OR divided by the geometric mean of all ORs. This measure can be interpreted as the OR for a comparison between a given county and an “average” county.

#### Potential impact fractions

To determine the shares of deaths and hospitalizations that could potentially have been avoided if vaccination coverage in each county matched that in the most successful one within each stratum with respect to birth cohort and sex, we calculated PIFs [25] by outcome and period. To avoid sparse data on exposure distributions, these calculations were based on the vaccination exposure distributions in the full cohort, stratified by birth cohort, sex, and month only. Moreover, we defined vaccination exposure by the binary indicator “not vaccinated within the past 7–179 days” only. Reducing the share of individuals not vaccinated within the past 7–179 days is a well-defined and realistic target for public policy, and mirrors the pace of the COVID-19 vaccine rollouts in Sweden, which occurred at approximately 6-month intervals. Details of the PIF calculations are provided in Supplementary materials (Text S1). PIFs were calculated for each period, both for each county separately and for Sweden as a whole.

In turn, attributable mortality rates (AMRs) and attributable hospitalization rates (AHRs) were obtained by multiplying the PIFs by the total number of deaths or hospitalizations, respectively, and dividing by mid-year population sizes.

## Results

### Descriptives

**Figure 1.**
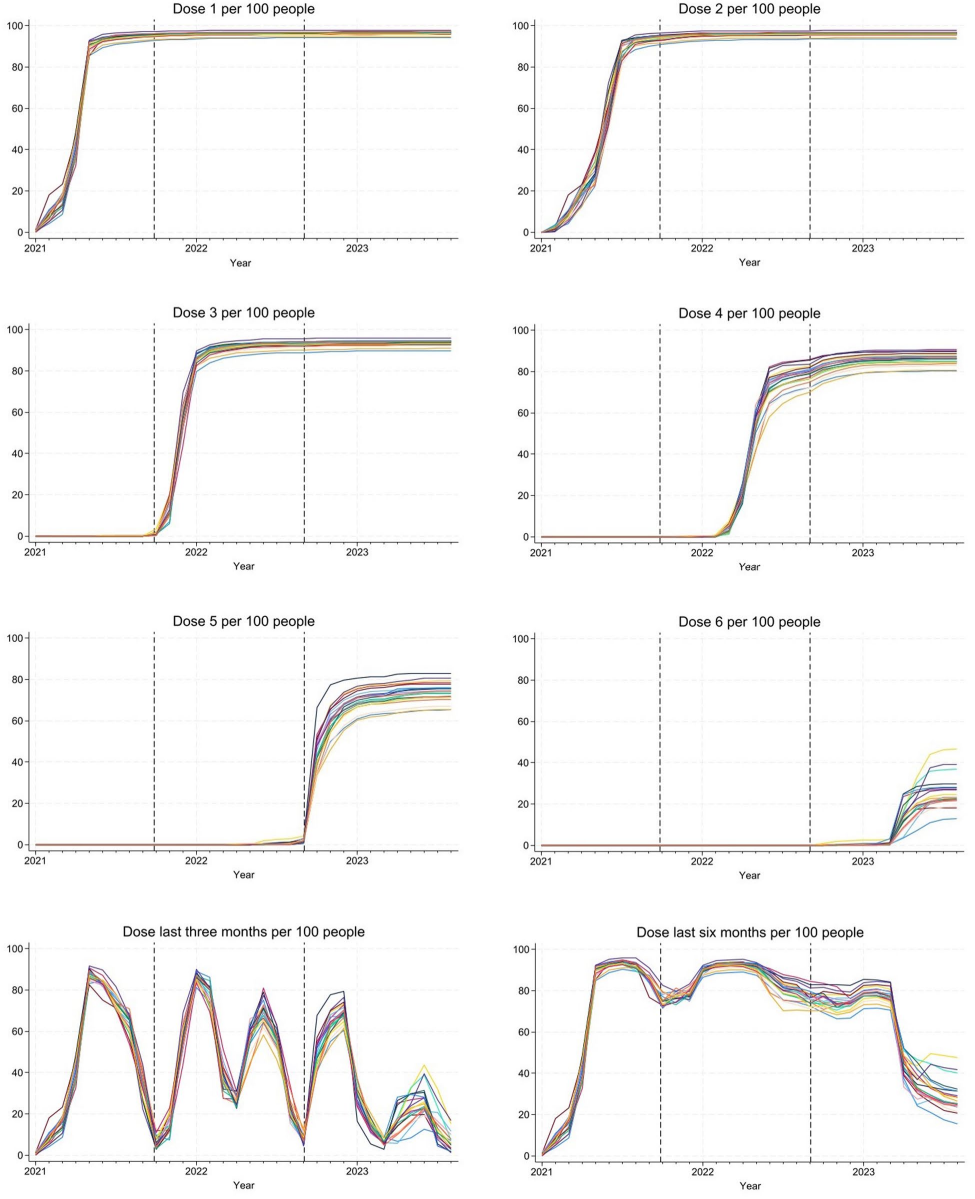
Vaccination coverage over time (whether having received Doses 1–6, and whether having received at least one dose during the last 3 or 6 months). Each colored line represents a county. The different study periods are separated by dashed lines. Descriptives are based on the full cohort.

Our full cohort included 2,080,104 individuals, distributed over 21 counties. Vaccination coverage over time is shown in Figure 1. The coverage of the first two doses was relatively similar across counties. Larger differences occurred for subsequent doses, especially toward the ends of the rollouts. Noticeable variations in the shares of individuals covered by any vaccination dose during the past 3 or 6 months were also detected, especially so during the summer and fall of 2023.

More detailed figures, allowing for particular counties to be discerned, are shown in Supplementary Figures S1–S8. Overall, coverage was comparatively low in the urban counties of Stockholm, Västra Götaland, and Skåne, and higher in more rural counties, such as Gotland and Värmland.

Limiting attention to the mortality controls (Supplementary Figure S9), hence keeping the distributions of age and sex identical to those in the cases, yielded similar patterns of vaccination coverage as for the full cohort.

Both mortality and hospitalizations were concentrated to early 2021, with smaller peaks occurring in the beginning of 2022 and around the turn of 2022/2023 (Supplementary Figure S10).

Background characteristics for the case-control samples are reported separately for cases (dead and hospitalized) and controls (Supplementary Tables S1 and S2). There were 4393 deaths in Period 1 (matched to 43,922 controls), 2571 in Period 2 (25,690 controls), and 2129 in Period 3 (21,276 controls). There were 11,972 hospitalizations in Period 1 (119,661 controls), 11,737 in Period 2 (117,310 controls), and 11,067 in Period 3 (110,627 controls). In addition to differences with respect to county of residence, cases were more likely than controls to be born abroad, less likely to be married/registered partners, more likely to reside in nursing homes or to receive home care, more likely to have comorbidities, less likely to be recently vaccinated, and more likely to be unvaccinated.

### Outcome associations and county disparities

Models 1 and 2 regression results revealed that COVID-19 mortality (Table I) and hospitalizations (Supplementary Table S3) were both strongly associated with the presence of comorbidities and whether the individual received home assistance or was a nursing home resident.

**Table I.** COVID-19 mortality ratios.

	Period 1		Period 2		Period 3	
Mortality ratios (95% CI)	Model 1	Model 2	Model 1	Model 2	Model 1	Model 2
Foreign-born	1.50 (1.36–1.65)	1.44 (1.29–1.59)	1.51 (1.32–1.72)	1.10 (0.95–1.27)	1.06 (0.91–1.25)	0.86 (0.73–1.02)
Married/registered partner	0.99 (0.91–1.07)	1.01 (0.93–1.10)	0.98 (0.88–1.09)	1.11 (0.99–1.25)	1.13 (1.00–1.27)	1.25 (1.10–1.41)
Nursing home resident	7.21 (6.44–8.09)	14.97 (13.12–17.09)	12.28 (10.57–14.25)	13.84 (11.79–16.24)	12.48 (10.61–14.69)	14.14 (11.96–16.73)
Receiving home assistance	2.49 (2.25–2.75)	3.11 (2.80–3.45)	4.06 (3.55–4.64)	4.03 (3.50–4.65)	4.47 (3.86–5.18)	4.43 (3.81–5.16)
Share secondary education	0.06 (0.03–0.13)	0.06 (0.03–0.14)	0.15 (0.05–0.41)	0.41 (0.13–1.26)	1.00 (0.30–3.36)	1.53 (0.44–5.30)
Share tertiary education	0.11 (0.06–0.19)	0.11 (0.06–0.20)	0.14 (0.06–0.29)	0.32 (0.15–0.72)	0.73 (0.30–1.78)	1.06 (0.43–2.64)
**Comorbidities**						
Cardiovascular disease	1.87 (1.72–2.04)	1.89 (1.73–2.06)	1.59 (1.42–1.78)	1.68 (1.50–1.90)	1.66 (1.47–1.88)	1.70 (1.50–1.93)
Diabetes or obesity	1.77 (1.63–1.93)	1.79 (1.63–1.95)	1.29 (1.15–1.45)	1.27 (1.12–1.44)	1.22 (1.07–1.38)	1.21 (1.06–1.39)
Kidney or liver disease	2.11 (1.87–2.38)	2.19 (1.93–2.48)	2.23 (1.91–2.59)	2.28 (1.94–2.68)	2.34 (1.98–2.77)	2.41 (2.03–2.87)
Respiratory disease	5.31 (4.91–5.75)	5.40 (4.97–5.87)	4.50 (4.04–5.01)	4.37 (3.89–4.91)	4.05 (3.60–4.56)	4.11 (3.64–4.65)
Neurological disease	1.76 (1.62–1.91)	1.83 (1.68–1.99)	1.60 (1.44–1.78)	1.66 (1.49–1.86)	1.75 (1.56–1.96)	1.80 (1.60–2.02)
Cancer/immunosuppressed	1.24 (1.14–1.35)	1.28 (1.17–1.40)	1.29 (1.15–1.44)	1.43 (1.28–1.61)	1.21 (1.07–1.36)	1.32 (1.16–1.49)
Other comorbidity	1.08 (0.95–1.24)	1.07 (0.94–1.23)	1.25 (1.04–1.49)	1.11 (0.91–1.35)	1.22 (1.01–1.48)	1.13 (0.93–1.39)
**Last vaccination**						
Last 3 months		0.55 (0.33–0.93)		0.47 (0.28–0.80)		0.41 (0.29–0.58)
3–6 months ago		0.39 (0.23–0.65)		0.84 (0.51–1.37)		0.72 (0.52–1.00)
6–9 months ago				1.58 (0.98–2.54)		1.00 (0.73–1.37)
9–12 months ago				1.62 (0.99–2.62)		0.97 (0.72–1.30)
Further back/never (ref.)		1.00		1.00		1.00
**Number of doses**						
0 (ref.)		1.00		1.00		1.00
1		0.75 (0.44–1.27)		0.39 (0.21–0.71)		0.65 (0.34–1.25)
2		0.11 (0.07–0.18)		0.25 (0.16–0.39)		0.30 (0.21–0.42)
3				0.13 (0.08–0.22)		0.26 (0.20–0.35)
4				0.17 (0.09–0.30)		0.24 (0.17–0.34)
5						0.23 (0.16–0.34)
6						0.21 (0.13–0.35)
*N*	48,316	48,316	28,261	28,261	23,405	23,405

*Note*: COVID-19 mortality ratios from Models 1 and 2, estimated with logistic regression on the case-control dataset on deaths. Period 1 covers the rollout of Doses 1 and 2 (January 1, 2021–September 27, 2021); Period 2 covers the rollout of Doses 3 and 4 (September 28, 2021–August 31, 2022); Period 3 covers the rollout of Doses 5 and 6 (September 1, 2022–August 31, 2023). Effect estimates for the highest category of “Number of doses” should be interpreted as “at least this number of doses” (at least two doses for Period 1, at least four doses for Period 2, and at least six doses for Period 3). Confidence intervals (95% CI) are given within parentheses. All models were additionally adjusted for county fixed effects (shown in Figure 2).

**Figure 2.**
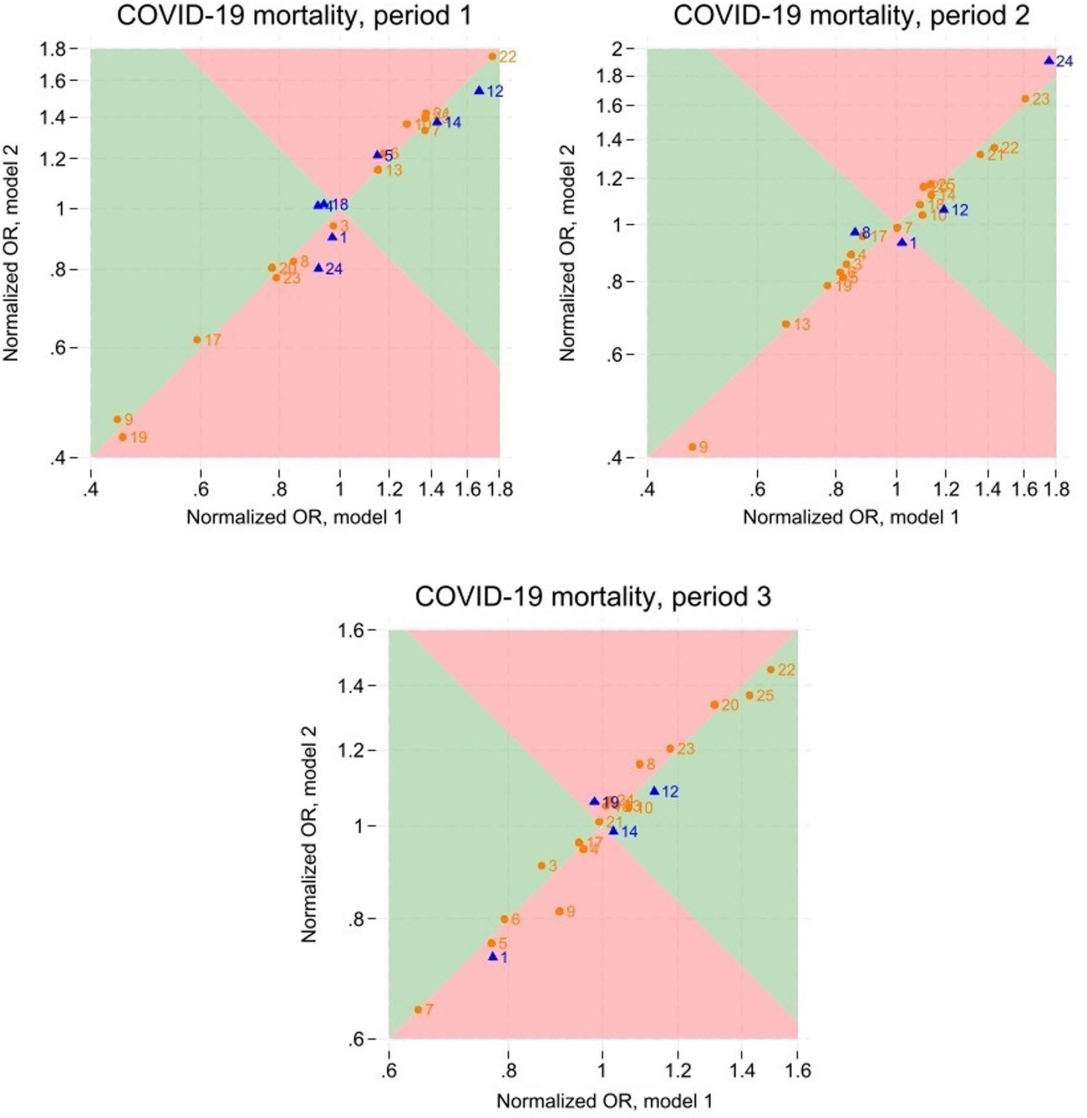
Normalized odds ratios for COVID-19 mortality from Models 1 and 2. The figure shows results for the three periods: Periods 1, 2, and 3. Period 1 covers the rollout of Doses 1 and 2 (January 1, 2021–September 27, 2021); Period 2 covers the rollout of Doses 3 and 4 (September 28, 2021–August 31, 2022); Period 3 covers the rollout of Doses 5 and 6 (September 1, 2022–August 31, 2023). The blue triangles represent counties for which the normalized odds ratios from the two models were statistically different at the 0.05 level; other counties are represented by orange circles. For counties in the green areas, estimates from Model 2 were less extreme than those from Model 1; for counties in the red areas, the opposite held. The numbers represent the official county numbers (indicated in Table II).

**Table II.** COVID-19 deaths, potential impact fractions PIFs, and AMRs.

	Period 1			Period 2			Period 3		
County	Actual deaths	PIF	AMR	Actual deaths	PIF	AMR	Actual deaths	PIF	AMR
1 Stockholm	858	0.11 (0.10–0.12)	25 (23–26)	530	0.15 (0.13–0.16)	22 (20–24)	359	0.18 (0.16–0.20)	18 (16–21)
3 Uppsala	128	0.08 (0.07–0.08)	14 (13–15)	66	0.06 (0.06–0.07)	6 (5–7)	61	0.04 (0.03–0.04)	4 (3–4)
4 Södermanland	106	0.04 (0.04–0.04)	6 (6–7)	75	0.10 (0.09–0.11)	12 (11–13)	68	0.12 (0.11–0.14)	14 (12–16)
5 Östergötland	208	0.03 (0.03–0.03)	7 (6–7)	101	0.05 (0.04–0.05)	6 (5–6)	84	0.09 (0.08–0.10)	9 (7–10)
6 Jönköping	176	0.06 (0.06–0.06)	14 (13–15)	81	0.06 (0.06–0.07)	7 (6–8)	84	0.08 (0.07–0.09)	10 (9–11)
7 Kronoberg	101	0.09 (0.09–0.10)	22 (21–23)	53	0.13 (0.11–0.14)	17 (15–18)	32	0.13 (0.11–0.14)	10 (9–12)
8 Kalmar	98	0.07 (0.07–0.08)	12 (11–12)	60	0.06 (0.05–0.07)	6 (5–7)	70	0.12 (0.10–0.13)	15 (13–17)
9 Gotland	11	0.04 (0.04–0.04)	3 (3–3)	7	0.10 (0.09–0.11)	5 (4–5)	14	0.11 (0.09–0.12)	10 (9–12)
10 Blekinge	77	0.04 (0.04–0.05)	9 (8–10)	42	0.09 (0.08–0.10)	11 (10–12)	42	0.12 (0.10–0.13)	14 (13–17)
12 Skåne	780	0.10 (0.09–0.11)	29 (27–30)	360	0.14 (0.12–0.15)	19 (17–21)	273	0.09 (0.08–0.10)	10 (9–12)
13 Halland	151	0.09 (0.08–0.10)	19 (18–20)	65	0.09 (0.08–0.10)	8 (8–9)	83	0.08 (0.07–0.09)	10 (9–12)
14 Västra Götaland	830	0.09 (0.08–0.09)	21 (20–23)	417	0.11 (0.10–0.13)	15 (13–17)	341	0.11 (0.09–0.13)	12 (11–14)
17 Värmland	78	0.04 (0.04–0.04)	5 (4–5)	69	0.02 (0.01–0.02)	2 (2–2)	72	0.01 (0.01–0.01)	1 (1–1)
18 Örebro	96	0.03 (0.02–0.03)	4 (4–5)	72	0.09 (0.08–0.09)	10 (9–11)	67	0.12 (0.11–0.14)	14 (12–15)
19 Västmanland	65	0.09 (0.08–0.10)	10 (9–10)	73	0.09 (0.09–0.10)	12 (11–13)	68	0.06 (0.05–0.06)	7 (6–8)
20 Dalarna	93	0.04 (0.03–0.04)	5 (5–6)	88	0.04 (0.04–0.05)	5 (5–6)	90	0.05 (0.05–0.06)	8 (6–9)
21 Gävleborg	155	0.06 (0.06–0.07)	15 (14–16)	96	0.10 (0.09–0.11)	15 (13–17)	76	0.10 (0.08–0.11)	12 (10–14)
22 Västernorrland	152	0.08 (0.07–0.08)	20 (18–21)	85	0.08 (0.07–0.09)	13 (11–14)	74	0.12 (0.11–0.14)	17 (15–20)
23 Jämtland	32	0.07 (0.06–0.07)	7 (6–8)	46	0.09 (0.09–0.10)	15 (13–17)	28	0.14 (0.12–0.15)	14 (12–16)
24 Västerbotten	82	0.11 (0.10–0.12)	15 (14–16)	101	0.01 (0.01–0.01)	2 (2–2)	59	0.06 (0.05–0.06)	6 (5–7)
25 Norrbotten	133	0.09 (0.08–0.10)	20 (19–22)	75	0.07 (0.07–0.08)	10 (9–11)	87	0.13 (0.11–0.15)	21 (18–23)
Overall	4410	0.08 (0.08–0.09)	18 (17–19)	2562	0.10 (0.09–0.11)	13 (12–15)	2132	0.11 (0.09–0.12)	12 (11–14)

*Note*: Actual deaths, potential impact fractions (PIFs), and attributable mortality rates (AMRs) per 100,000 individuals (AMR) for COVID-19 mortality. Normal-based 95% bootstrap confidence intervals for PIF and AMR are given within parentheses. Period 1 covers the rollout of Doses 1 and 2 (January 1, 2021–September 27, 2021); Period 2 covers the rollout of Doses 3 and 4 (September 28, 2021–August 31, 2022); Period 3 covers the rollout of Doses 5 and 6 (September 1, 2022–August 31, 2023).

Model 2 additionally revealed substantial protective effects of the vaccinations, both in terms of time since last dose and total number of doses. Conditional on number of doses, vaccination in the past 1–3 and 4–6 months reduced mortality by factors of 0.55 (95% CI 0.33–0.93) and 0.39 (95% CI 0.23–0.65) in Period 1; 0.47 (95% CI 0.28–0.80) and 0.84 (95% CI 0.51–1.37) in Period 2; and 0.41 (95% CI 0.29–0.58) and 0.72 (95% CI 0.52–1.00) in Period 3, as compared to the reference groups. Conditional on time since the last dose, having received two doses reduced mortality by a factor of 0.11 (95% CI 0.07–0.18) in Period 1; 0.25 (95% CI 0.16–0.29) in Period 2; and 0.30 (95% CI 0.21–0.42) in Period 3. Doses beyond the first two offered no clear additional protection, conditional on time since the last dose. Vaccine effects on COVID-19 hospitalizations followed broadly similar patterns as mortality.

There were substantial disparities in outcome incidences across counties. Compared to the average county, mortality (Figure 2; Supplementary Figure S11) varied from −56% to +75% in Period 1, from −53% to +75% in Period 2, and from −36% to +50% in Period 3, according to Model 1 estimates. The corresponding ranges for hospitalizations (Supplementary Figures S12 and S13) were from −45% to +38% in Period 1, from −28% to +49% in Period 2, and from −23% to +51% in Period 3. Model 2 estimates of county disparities, additionally adjusted for vaccinations, resembled those from Model 1. For mortality, the ORs changed by no more than 13% upon this additional adjustment, whereas for hospitalizations, changes never exceeded 7%. The direction of the changes displayed no consistent pattern, with some becoming more extreme, others less.

### Potential impact fractions

The PIF for mortality (Table II) varied from 0.03 to 0.11 across counties in Period 1, from 0.02 to 0.15 in Period 2, and from 0.01 to 0.18 in Period 3, with overall PIFs amounting to 0.08, 0.10, and 0.11 in the three periods, respectively. The overall AMR was 18, 13, and 12 per 100,000 people in Periods 1, 2, and 3. For hospitalizations (Supplementary Table S4), PIF ranged from 0.03 to 0.09 in Period 1, from 0.01 to 0.12 in Period 2, and from 0.01 to 0.13 in Period 3, with overall PIFs of 0.07, 0.08, and 0.08. The overall AHR was 40, 47, and 48 per 100,000 people across periods. Broadly mirroring the previously documented discrepancies in vaccination coverage, the potential for reducing COVID-19 deaths and hospitalizations tended to be largest in urban counties such as Stockholm, and smaller in rural counties, particularly Värmland.

## Discussion

This study is one of the few to examine the extent to which regional disparities in COVID-19 outcomes could be explained by disparities in COVID-19 vaccination coverage. For both COVID-19 mortality and hospitalizations among older people in Sweden, we found substantial disparities across counties during the study period. However, while vaccines were highly effective, accounting for differences in vaccination coverage had little impact on the disparities in the outcomes, regardless of the period. Mirroring the small changes in the county effects upon vaccination adjustment, the PIFs were relatively low, suggesting that a hypothetically more effective vaccination rollout would have had a somewhat limited impact on reducing COVID-19 mortality and hospitalization.

While the limited role played by regional differences in vaccination coverage might seem somewhat at odds with the heterogeneous coverage of vaccinations that we have documented, especially of the last few vaccine doses, two things need to be noted. First, what matters is not the eventual disparities in vaccination coverage, but rather the disparities that prevailed when the outcomes occurred. Second, beyond the initial two doses, what matters for the outcomes is not how many doses an individual has received, but rather the time since the last dose, a phenomenon that has also been previously documented [26]. Considering the share of the population vaccinated within the past 3 or 6 months (Figure 1; Supplementary Figures S7 and S8) during the times when most outcomes occurred (Supplementary Figure S10), county disparities were rather limited. We conclude that the Swedish counties were relatively successful in their vaccination rollouts at the times when coverage mattered most. Moreover, while county disparities in vaccination coverage have tended to be greater in more recent time periods, the protective effects of the vaccines have also tended to become weaker. These two phenomena have counteracting effects on the county disparities in the outcomes, leading to relatively similar impacts of vaccination uptake on the disparities in COVID-19 outcomes across the three time periods.

In addition to vaccinations, regional disparities in COVID-19 outcomes may reflect a range of individual- and contextual-level factors. Studies from Sweden in 2020 have pointed to factors such as population density, the share of immigrants, age structure [6], and proximity to areas with more infections [7]. Future studies should explore the extent to which these patterns persist into the post-vaccination period.

Our study has both strengths and limitations. Among the strengths, we were able to improve upon previous literature on the association between COVID-19 vaccination and regional outcomes by utilizing large-scale, individual-level data on vaccinations and outcomes, and adjusting for a large set of sociodemographic factors and comorbidities, as well for all additional unobserved factors at the aggregate level—as captured by the county fixed effects. While our findings provide insights suggesting that regional disparities in the rollout and uptake of vaccinations had little impact on regional disparities in pandemic outcomes in Sweden, it is important to note that this result is not necessarily generalizable to other settings. In Germany [27] and the United States [16], for example, COVID-19 vaccination coverage varied quite substantially across regions, and it is possible that in these countries, differences in vaccination coverage could explain a larger part of the regional differences in pandemic outcomes.

Among the limitations of our study, there may be unobserved confounding factors such as unregistered health conditions or health-related behaviors such as social distancing and adherence to preventive measures, as well as socioeconomic factors beyond area-level education, which were not taken into account by our regression models. Another limitation is that we only captured the direct protective effects of the vaccines on the vaccinated individual, without considering the indirect effects on others. Vaccination can also prevent the spread of disease [28], meaning that we may have underestimated the total role of vaccinations in shaping regional differences. However, as our analysis suggested no substantial role of direct vaccine effects in shaping regional differences, it is unlikely that indirect effects would play a major role.

## Conclusion

Regional disparities in the coverage of COVID-19 vaccination were too small to substantially shape regional disparities in COVID-19 mortality and COVID-19 hospitalizations among older people in Sweden during 2021–2023. Nevertheless, higher vaccination coverage particularly in urban areas such as Stockholm County would have saved lives and avoided hospitalizations, underscoring that there are benefits to improved access to vaccination. Future investigations of regional disparities in pandemic or other health outcomes may use our approach to also investigate the role of factors other than vaccinations, including socioeconomic conditions, comorbidities, and access to healthcare or testing.

## Supplemental Material

sj-docx-1-sjp-10.1177_14034948261420643 – Supplemental material for COVID-19 vaccination coverage and the effect on regional disparities in morbidity and mortality among older people in Sweden, 2021–2023 Supplemental material, sj-docx-1-sjp-10.1177_14034948261420643 for COVID-19 vaccination coverage and the effect on regional disparities in morbidity and mortality among older people in Sweden, 2021–2023 by Anton Nilsson, Dominik Dietler, Carl Bonander, Malin Inghammar and Jonas Björk in Scandinavian Journal of Public Health

## References

[1] Msemburi W Karlinsky A Knutson V , et al. The WHO estimates of excess mortality associated with the COVID-19 pandemic. Nature 2023;613:130–7. 10.1038/s41586-022-05522-2 PMC9812776 36517599

[2] Mathieu E Ritchie H Rodés-Guirao L , et al. Coronavirus pandemic (COVID-19), https://ourworldindata.org/coronavirus (2020, accessed 17 December 2023).

[3] Abu-Hammad O Alnazzawi A Borzangy S , et al. Factors influencing global variations in COVID-19 cases and fatalities: a review. Healthcare (Basel) 2020;8:216. 32708986 10.3390/healthcare8030216 PMC7551068

[4] Sorci G Faivre B Morand S. Explaining among-country variation in COVID-19 case fatality rate. Sci Rep 2020;10:18909. 33144595 10.1038/s41598-020-75848-2 PMC7609641

[5] Siddiqui SH Sarfraz A Rizvi A , et al. Global variation of COVID-19 mortality rates in the initial phase. Osong Public Health Res Perspect 2021;12:64–72. 33979996 10.24171/j.phrp.2021.12.2.03 PMC8102879

[6] Fonseca-Rodríguez O Gustafsson PE San Sebastián M , et al. Spatial clustering and contextual factors associated with hospitalisation and deaths due to COVID-19 in Sweden: a geospatial nationwide ecological study. BMJ Glob Health 2021;6:e006247. 10.1136/bmjgh-2021-006247 PMC8322019 34321234

[7] Florida R Mellander C. The geography of COVID-19 in Sweden. Ann Reg Sci 2022;68:125–50. 10.1007/s00168-021-01071-0 PMC8299438 34316091

[8] Hajdu T Krekó J Tóth CG. Inequalities in regional excess mortality and life expectancy during the COVID-19 pandemic in Europe. Sci Rep 2024;14:3835. 38360870 10.1038/s41598-024-54366-5 PMC10869827

[9] Bonnet F Grigoriev P Sauerberg M , et al. Spatial variation in excess mortality across Europe: a cross-sectional study of 561 regions in 21 countries. J Epidemiol Glob Health 2024;14:470–9. 10.1007/s44197-024-00200-0 PMC11176282 38376764

[10] Bonnet F Grigoriev P Sauerberg M , et al. Spatial disparities in the mortality burden of the COVID-19 pandemic across 569 European regions (2020–2021). Nat Commun 2024;15:4246. 38762653 10.1038/s41467-024-48689-0 PMC11102496

[11] Bourdin S Levratto N. Regional implications of COVID-19. Int Reg Sci Rev 2023;46:515–22.

[12] European Commission. Cohesion in Europe towards 2050: Eighth report on economic, social and territorial cohesion. Luxembourg: Publications Office of the European Union; 2022. https://ec.europa.eu/regional_policy/information-sources/8cohesion-report_en 2026-01-30.

[13] Friedman J Balaj M Islam N , et al. Inequalities in COVID-19 mortality: defining a global research agenda. Bull World Health Organ 2022;100:648–50. 10.2471/BLT.22.288211 PMC9511668 36188017

[14] Cuadros DF Moreno CM Musuka G , et al. Association between vaccination coverage disparity and the dynamics of the COVID-19 delta and omicron waves in the US. Front Med (Lausanne) 2022;9:898101. 35775002 10.3389/fmed.2022.898101 PMC9237603

[15] Cuadros DF Miller FD Awad S , et al. Analysis of vaccination rates and new COVID-19 infections by US county, July–August 2021. JAMA Netw Open 2022;5:e2147915. 10.1001/jamanetworkopen.2021.47915 PMC8832175 35142835

[16] McLaughlin JM Khan F Pugh S , et al. County-level vaccination coverage and rates of COVID-19 cases and deaths in the United States: an ecological analysis. Lancet Reg Health Am 2022;9:100191. 35128511 10.1016/j.lana.2022.100191 PMC8802692

[17] Pizzato M Gerli AG La Vecchia C , et al. Impact of COVID-19 on total excess mortality and geographic disparities in Europe, 2020–2023: a spatio-temporal analysis. Lancet Reg Health Eur 2024;44:100996. 39410937 10.1016/j.lanepe.2024.100996 PMC11473197

[18] Björk J Modig K Kahn F , et al. Revival of ecological studies during the COVID-19 pandemic. Eur J Epidemiol 2021;36:1225–9. 10.1007/s10654-021-00830-9 PMC8703212 34951671

[19] Janlöv N Blume S Glenngård AH , et al. Sweden: health system review. Health Syst Transit 2023;25:1–236. 38230685

[20] Xu Y Nyberg F Santosa A , et al. Regional differences in COVID-19 vaccine uptake and their determinants among Swedish older adults. Public Health 2025;24:324–31. 10.1016/j.puhe.2025.03.028 40179817

[21] Marking U. Folkhälsomyndighetens återrapportering av regeringsuppdrag: Uppdrag att undersöka orsaker till skillnader avseende andelen äldre personer som har vaccinerats mot covid-19 [The Public Health Agency’s report on government assignments: assignment to investigate the causes of differences in the proportion of older people who have been vaccinated against COVID-19]. Solna: Sweden, 2024.

[22] Dietler D Kahn F Inghammar M , et al. Waning protection after vaccination and prior infection against COVID-19-related mortality over 18 months. Clin Microbiol Infect 2023;29:1573–80. 10.1016/j.cmi.2023.08.007 37580016

[23] Björk J Bonander C Moghaddassi M , et al. COVID-19 vaccine effectiveness against severe disease from SARS-CoV-2 Omicron BA.1 and BA.2 subvariants – surveillance results from southern Sweden, December 2021 to March 2022. Euro Surveill 2022;27:2200322. 35514304 10.2807/1560-7917.ES.2022.27.18.2200322 PMC9074397

[24] Clayton DG Hills M. Statistical models in epidemiology. Oxford: Oxford University Press, 1993.

[25] Khosravi A Nazemipour M Shinozaki T , et al. Population attributable fraction in textbooks: time to revise. Glob Epidemiol 2021;3:100062. 37635714 10.1016/j.gloepi.2021.100062 PMC10445975

[26] Björk J Dietler D Bonander C , et al. Vaccine protection against COVID-19 mortality among nursing home residents in relation to time since last booster dose – a case-control study over 35 months. Vaccine 2025;71:128043. 41371095 10.1016/j.vaccine.2025.128043

[27] Bade V Schmitz H Tawiah BB. Regional variations in vaccination against COVID-19 in Germany. PLoS One 2024;19:e0296976. 10.1371/journal.pone.0296976 PMC11025766 38635523

[28] Oordt-Speets A Spinardi J Mendoza C , et al. Effectiveness of COVID-19 vaccination on transmission: a systematic review. COVID 2023;3:1516–27.

