# Supplementary material for: COVID-19 vaccination coverage and the effect on regional disparities in morbidity and mortality among older people in Sweden, 2021–2023
Source: Scand J Public Health. 2026 Feb 23;54(4):388–96. doi: 10.1177/14034948261420643 (PMC13176473; doi:10.1177/14034948261420643)
Supplement: sj-docx-1-sjp-10.1177_14034948261420643 – Supplemental material for COVID-19 vaccination coverage and the effect on regional disparities in morbidity and mortality among older people in Sweden, 2021–2023 [file sj-docx-1-sjp-10.1177_14034948261420643.docx]

**Supplement to *COVID-19 Vaccination Coverage and the Effect on Regional Disparities in Morbidity and Mortality among Older People in Sweden 2021 – 2023 (Scandinavian Journal of Public Health)***

Anton Nilsson^1.2,*^, Dominik Dietler^1^, Carl Bonander^3^, Malin Inghammar^4,5^, Jonas Björk^1,6^

^1^ Epidemiology, Population Studies and Infrastructures (EPI@LUND), Division of Occupational and Environmental Medicine, Epidemiology, Lund University, Lund, Sweden.

^2^ Register-based Epidemiology, Department of Translational Medicine, Lund University, Malmö, Sweden.

^3^ School of Public Health and Community Medicine, Institute of Medicine, University of Gothenburg, Gothenburg, Sweden

^4^ Infection Medicine, Department of Clinical Sciences Lund, Lund University, Lund, Sweden

^5^ Department of Infectious Diseases, Skåne University Hospital, Lund, Sweden

^6^ Clinical Studies Sweden, Forum South, Skåne University Hospital, Lund, Sweden

* Corresponding author. Email: [anton.nilsson@med.lu.se](mailto:anton.nilsson@med.lu.se)

**Contents**

[**Text S1:** Potential Impact Fractions 2](#_Toc198207673)

[**Fig S1.** Coverage of vaccination dose 1, across counties 3](#_Toc198207674)

[**Fig S2.** Coverage of vaccination dose 2, across counties 3](#_Toc198207675)

[**Fig S3**. Coverage of vaccination dose 3, across counties 4](#_Toc198207676)

[**Fig S4.** Coverage of vaccination dose 4, across counties 4](#_Toc198207677)

[**Fig S5.** Coverage of vaccination dose 5, across counties 5](#_Toc198207678)

[**Fig S6.** Coverage of vaccination dose 6, across counties 5](#_Toc198207679)

[**Fig S7.** Coverage of vaccination within the last three months, across counties 6](#_Toc198207680)

[**Fig S8.** Coverage of vaccination within the last six months, across counties 6](#_Toc198207681)

[**Fig S9.** Vaccination coverage over time among the controls 7](#_Toc198207682)

[**Fig S10.** COVID-19 deaths and hospitalizations over time 8](#_Toc198207683)

[**Fig S11.** Relative COVID-19 mortality across Swedish counties 9](#_Toc198207684)

[**Fig S12**. Normalized odds ratios for COVID-19 hospitalization from Models 1 and 2. 10](#_Toc198207685)

[**Fig S13.** Relative COVID-19 hospitalizations across Swedish counties 11](#_Toc198207686)

[**Table S1.** Descriptive statistics (COVID-19 mortality) 12](#_Toc198207687)

[**Table S2.** Descriptive statistics (COVID-19 hospitalizations) 14](#_Toc198207688)

[**Table S3.** COVID-19 hospitalization ratios 16](#_Toc198207689)

[**Table S4.** COVID-19 hospitalizations, PIF, and AHR 17](#_Toc198207690)

# **Text S1:** Potential Impact Fractions

As a first step, a PIF was calculated for each county, *c*, birth cohort, *b*, and sex, *s*:

1. ${PIF}_{c,b,s}=\sum_{m} \frac{q_{c,b,s,m}}{q_{c,b,s}}{PIF}_{c,b,s,m}=\sum_{m} \frac{q_{c,b,s,m}}{q_{c,b,s}}\frac{{(p}_{c,b,s,m}-p_{c,b,s,m}^{*})(OR-1)}{p_{c,b,s,m}\left( OR-1 \right)+1}$

The index *m* represents month; *q* is the number of events, *p* the share exposed (i.e., not vaccinated within the past 7-179 days), *p** a counterfactual share exposed, and *OR* is the odds ratio for the exposure effect, obtained from the case-control data. The *OR* is obtained from a regression where vaccination exposure is measured only with the above-mentioned binary indicator.

For each county, birth cohort, sex, and month, the counterfactual exposure share $p_{c,b,s,m}^{*}$ was taken to equal the exposure share $p_{c'(c,b,s),b,s,m}$in the same birth cohort, sex, and month in a (typically different) county $c'$, where $c'$ was selected for each combination of *c*, *b*, and *s* such as to maximize ${PIF}_{c,b,s}$.

Exposure shares were defined based on the 15^th^ day of each month. Birth cohorts were defined as ≤1930, 1931–1935, 1936–1940, 1941–1945, 1946–1950, and 1951–1955.

County-specific PIFs were subsequently calculated as:

1. ${PIF}_{c}=\sum_{b,s} \frac{q_{c,b,s}}{q_{c}}{PIF}_{c,b,s}$

And an overall PIF was obtained as:

1. $PIF=\sum_{c} \frac{q_{c}}{q}{PIF}_{c}$

Attributable Mortality Rates (AMR) and Attributable Hospitalization Rates (AHR) were obtained by multiplying PIFs by the number of cases and dividing by mid-year population sizes.

Considering the uncertainty in the estimation of the *ORs*, we calculated confidence intervals for the PIFs, AMRs, and AHRs using bootstrap applied to the case-control sample (1000 iterations, stratified on the matching units).


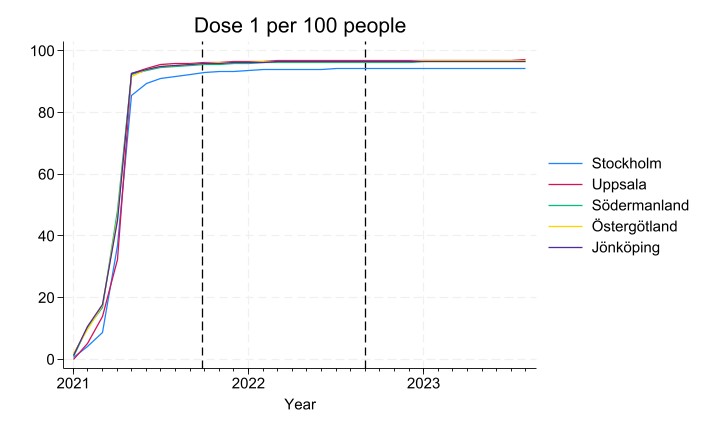

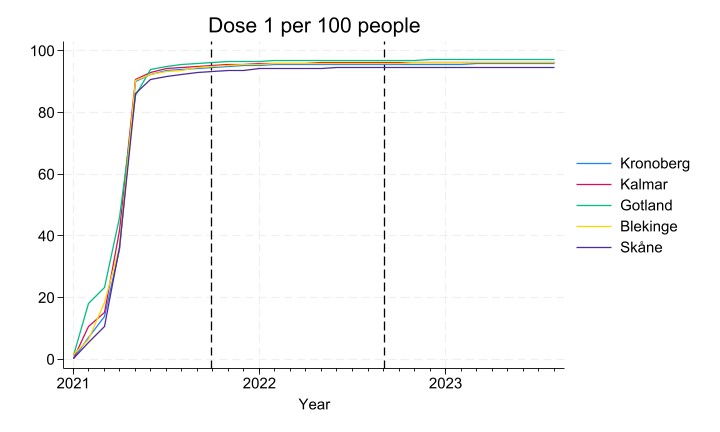

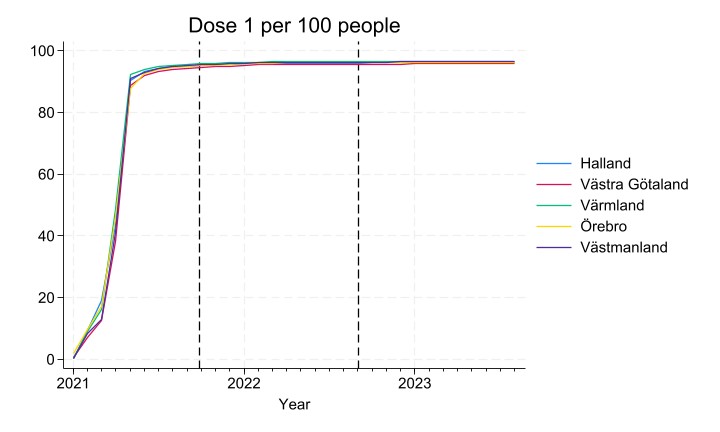

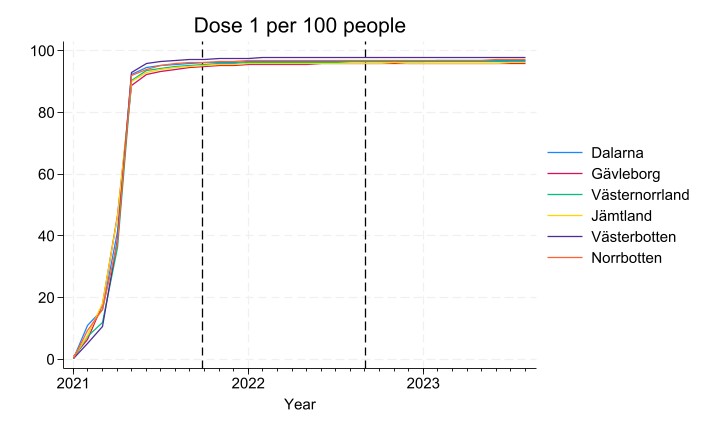


**Fig S1.** Coverage of vaccination dose 1, across counties. The dashed vertical lines indicate the start and end points of the three study periods. Data are based on the full cohort.


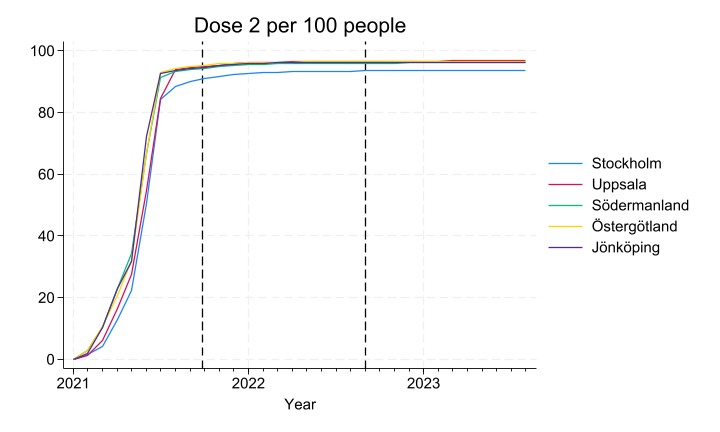

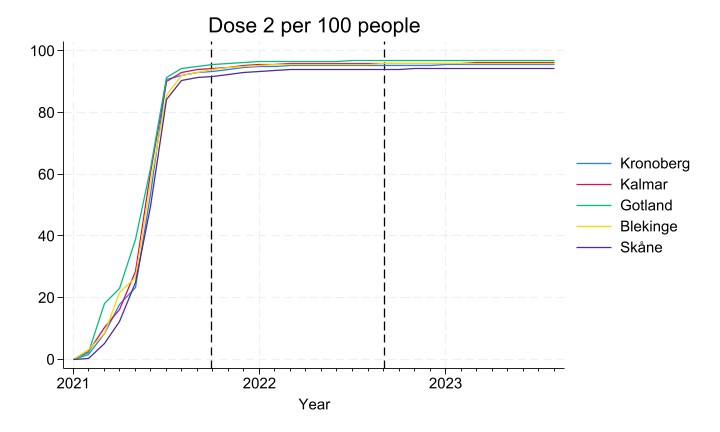

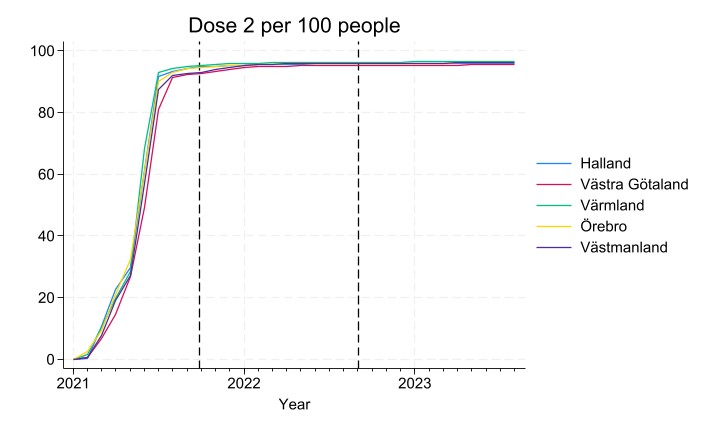

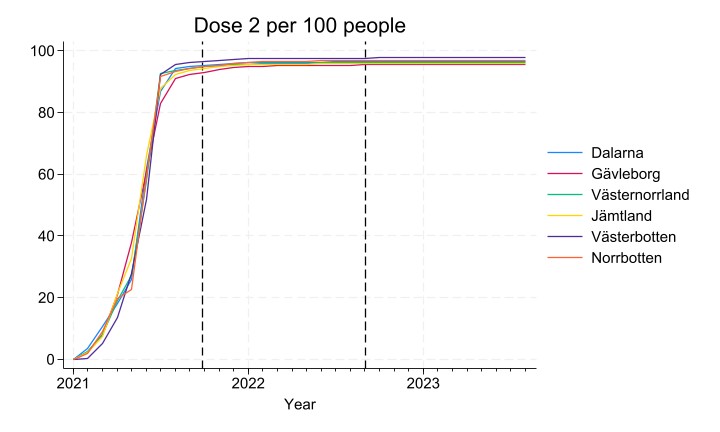


**Fig S2.** Coverage of vaccination dose 2, across counties. The different study periods are separated by dashed lines. Data are based on the full cohort.


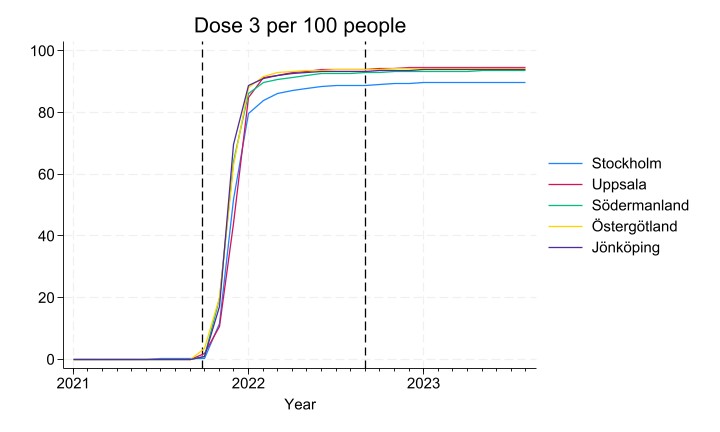

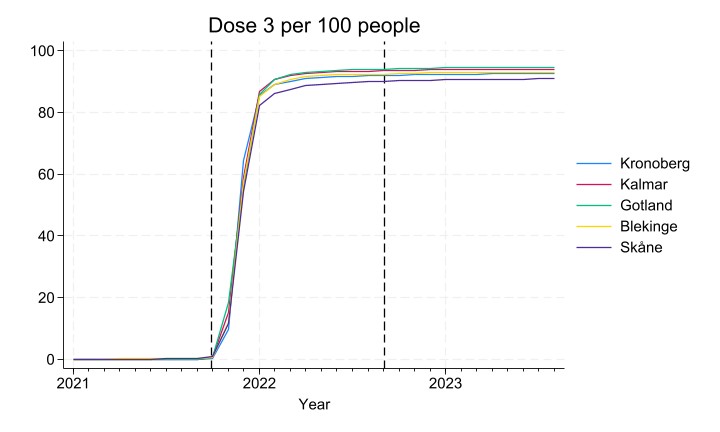

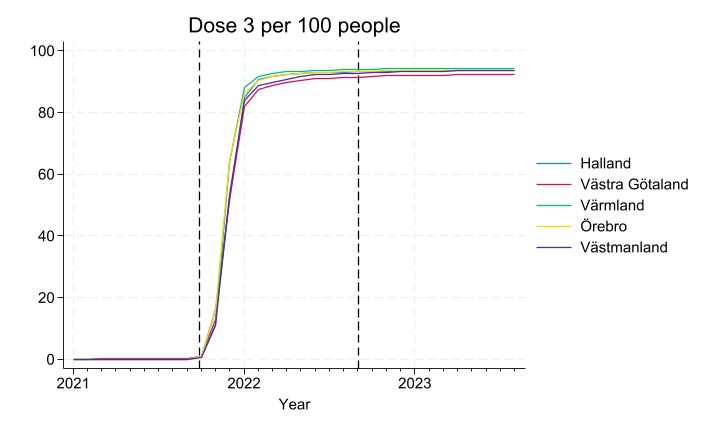

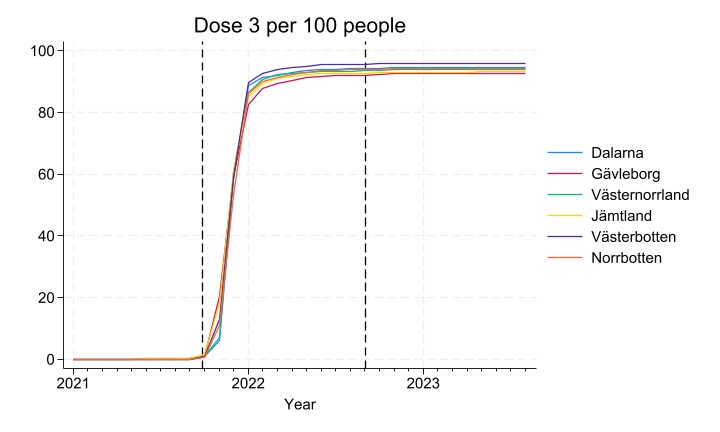


**Fig S3**. Coverage of vaccination dose 3, across counties. The different study periods are separated by dashed lines. Data are based on the full cohort.


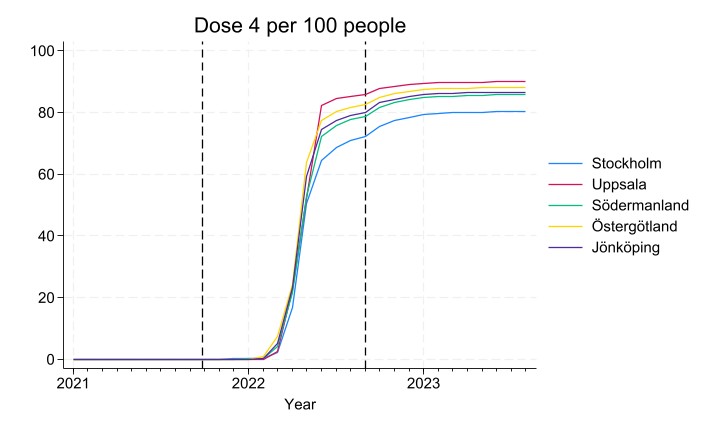

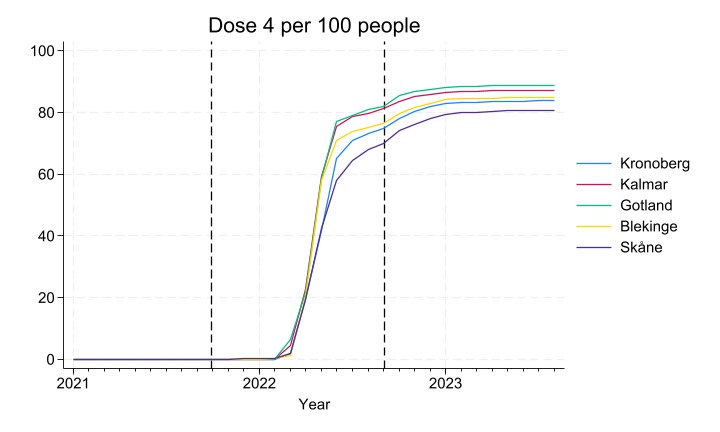

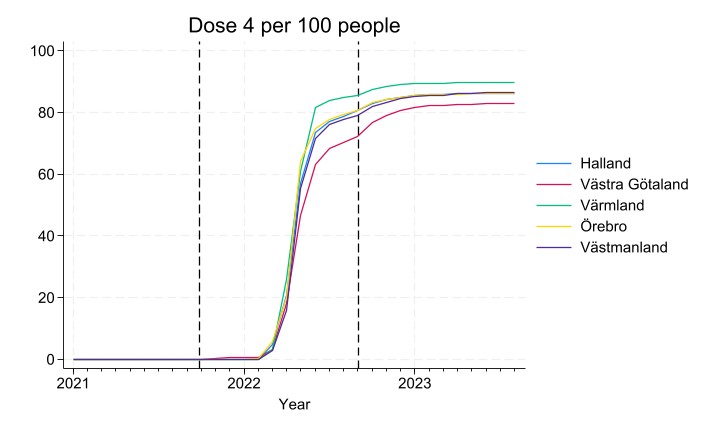

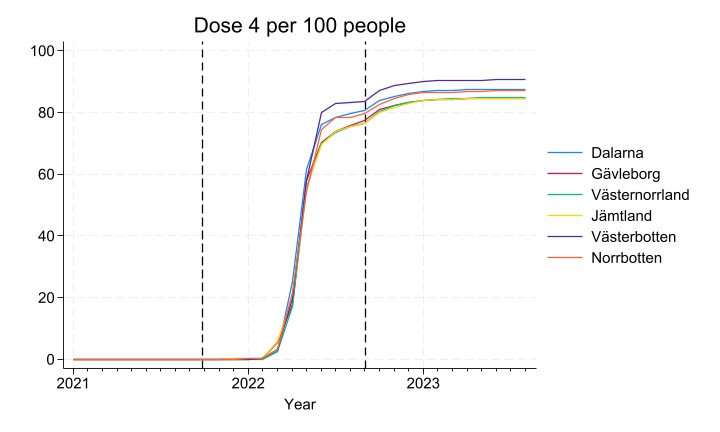


**Fig S4.** Coverage of vaccination dose 4, across counties. The different study periods are separated by dashed lines. Data are based on the full cohort.


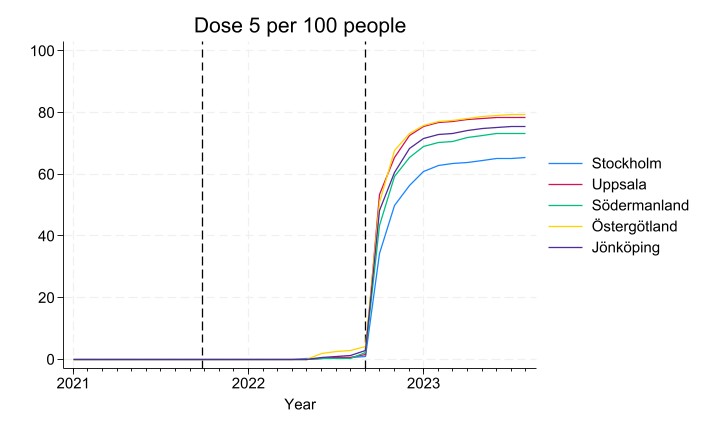

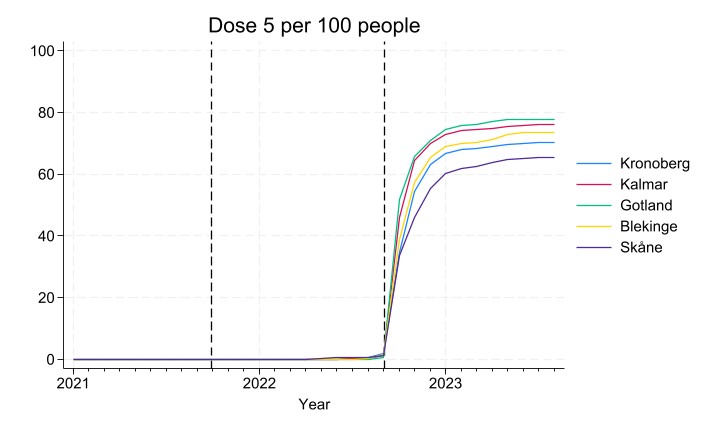

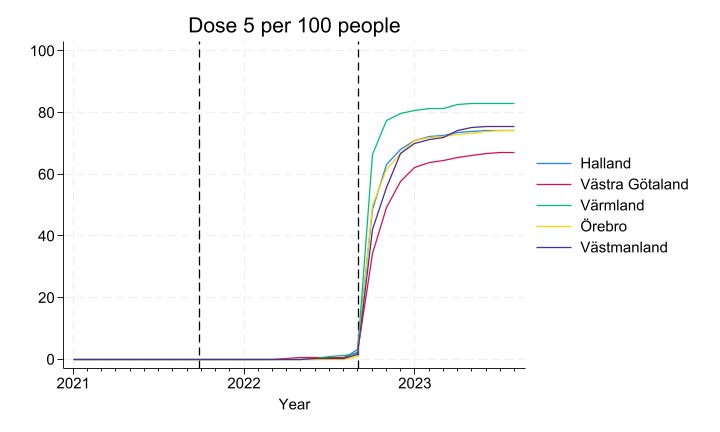

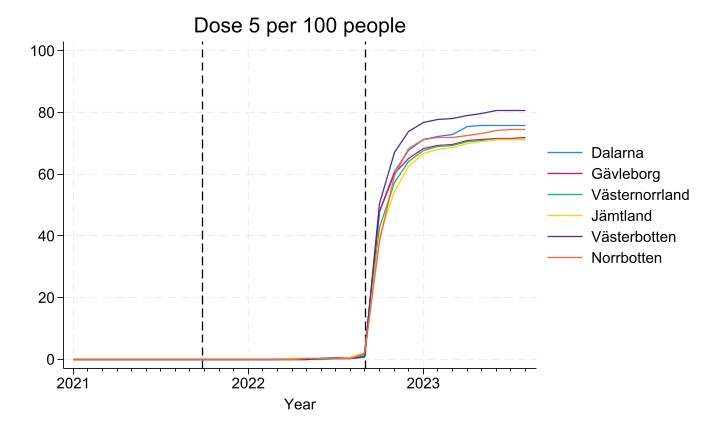


**Fig S5.** Coverage of vaccination dose 5, across counties. The different study periods are separated by dashed lines. Data are based on the full cohort.


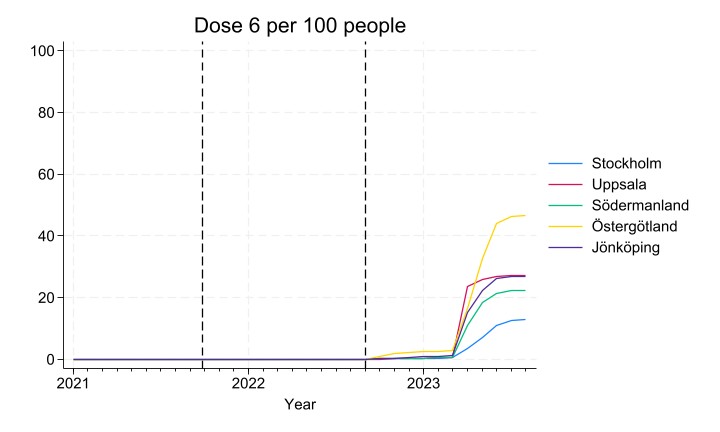

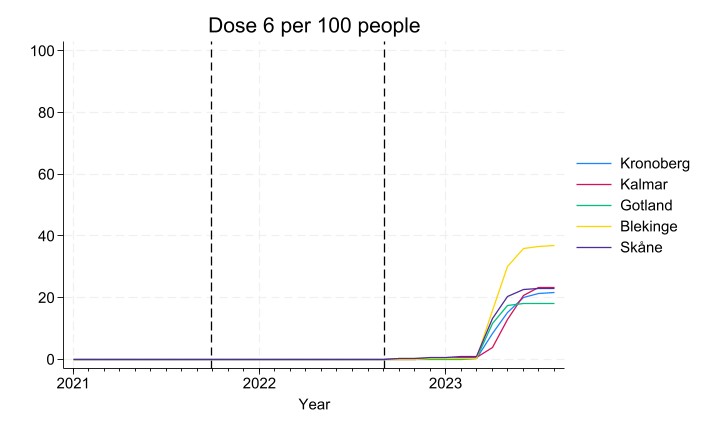

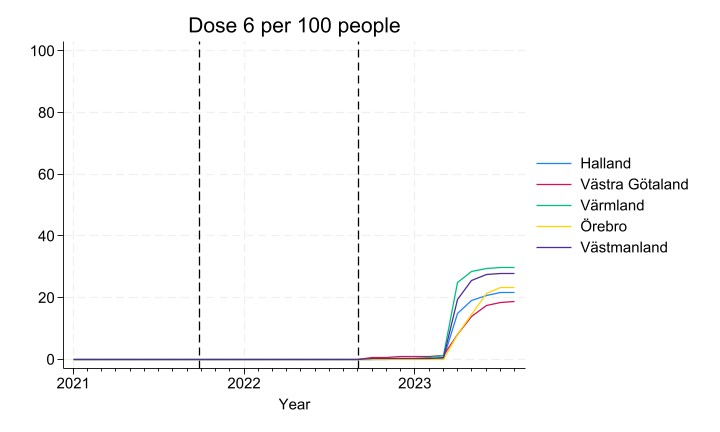

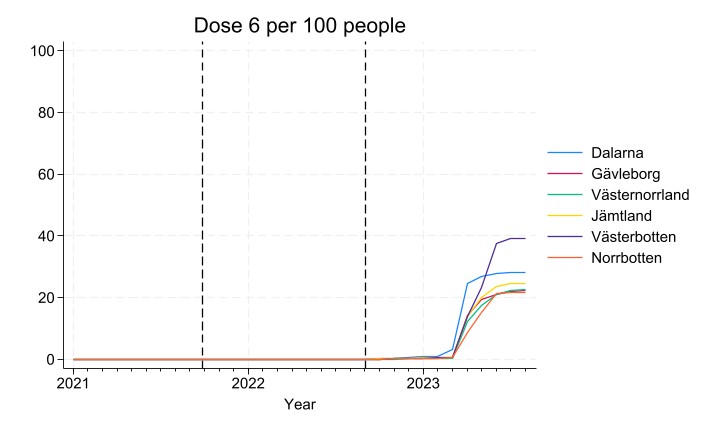


**Fig S6.** Coverage of vaccination dose 6, across counties. The different study periods are separated by dashed lines. Data are based on the full cohort.


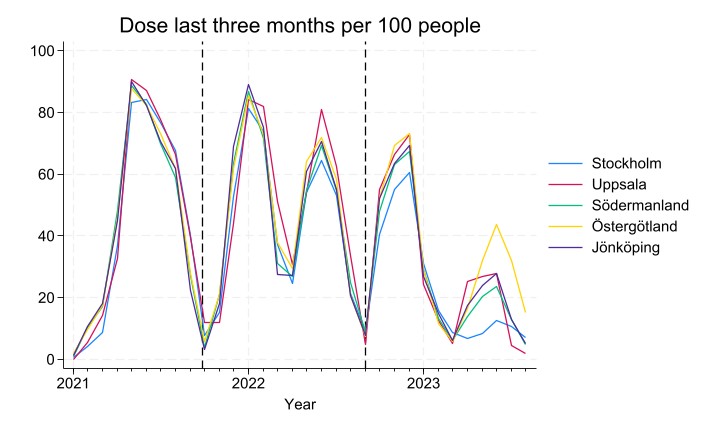

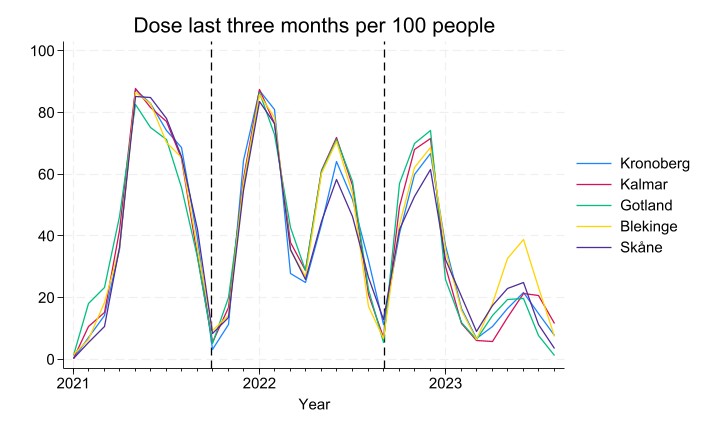

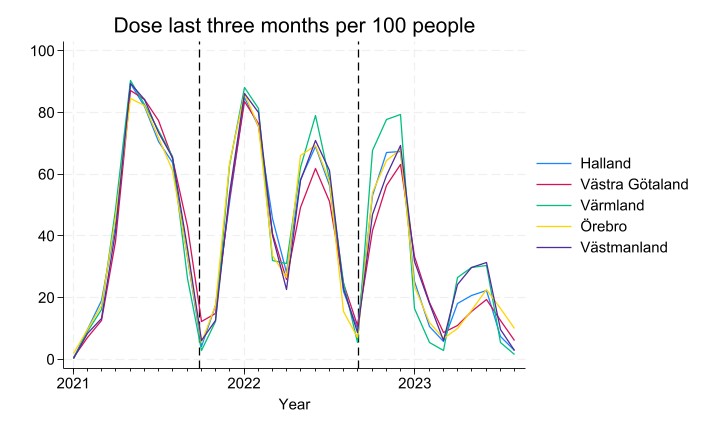

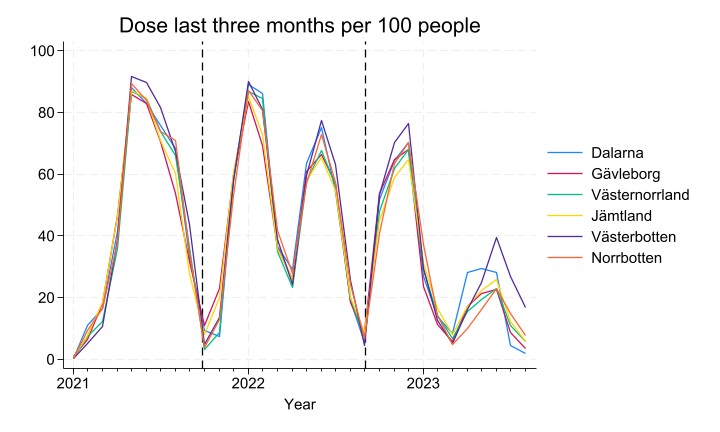


**Fig S7.** Coverage of vaccination within the last three months, across counties. The different study periods are separated by dashed lines. Data are based on the full cohort.


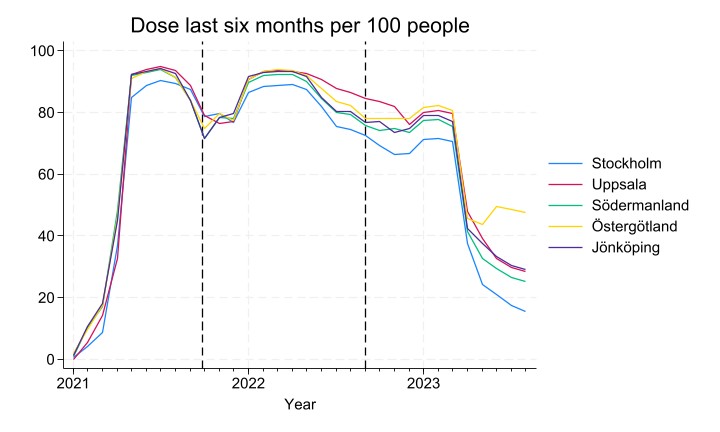

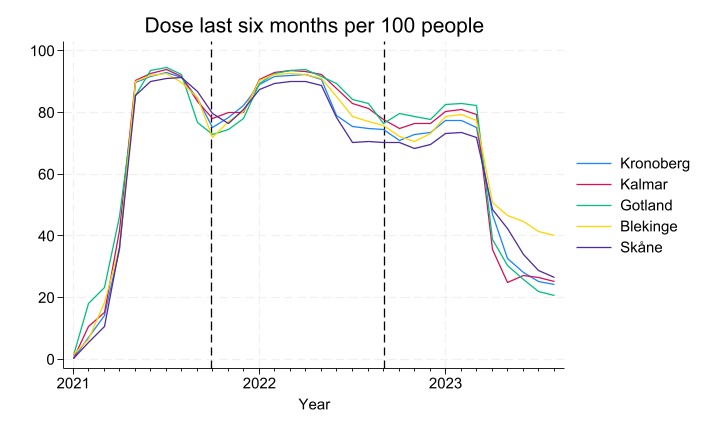

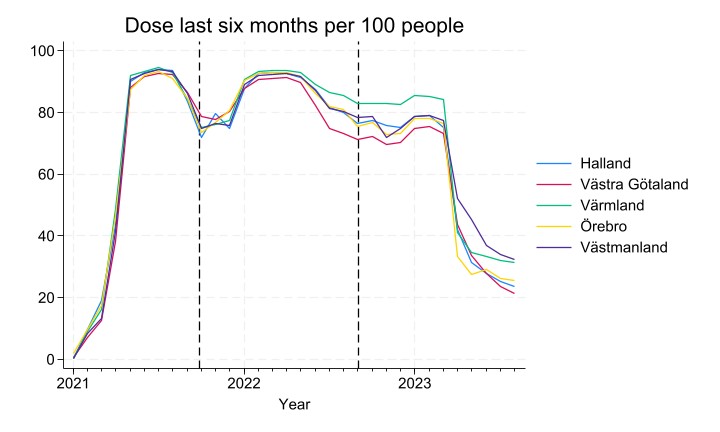

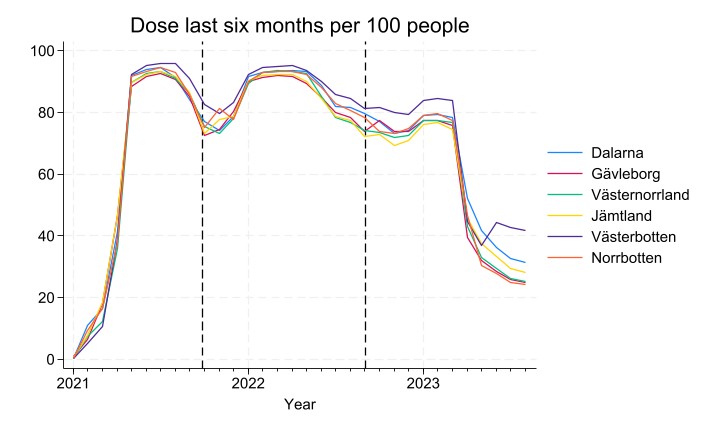


**Fig S8.** Coverage of vaccination within the last six months, across counties. The different study periods are separated by dashed lines. Data are based on the full cohort.


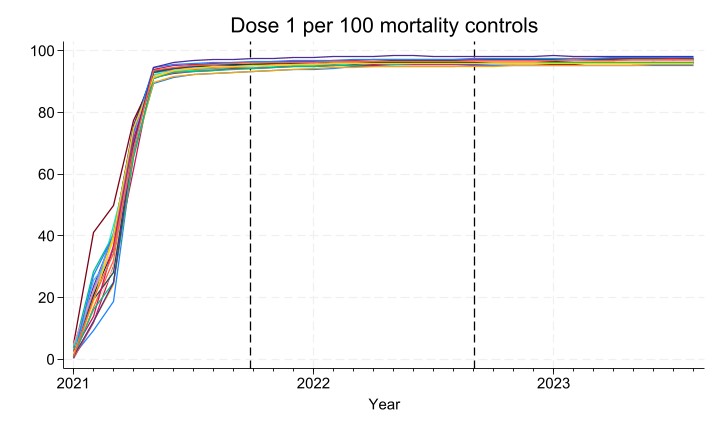

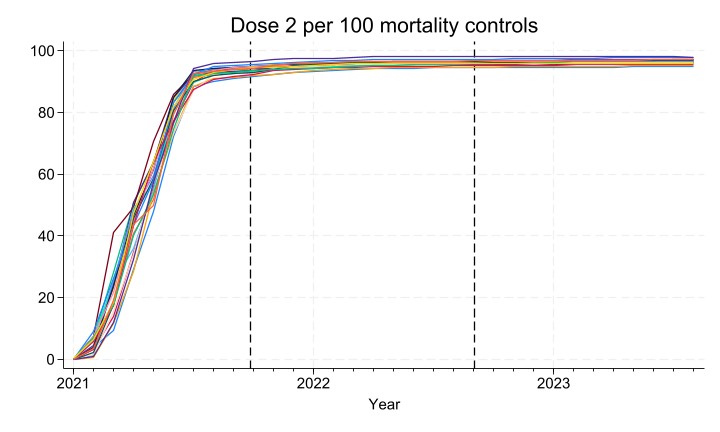

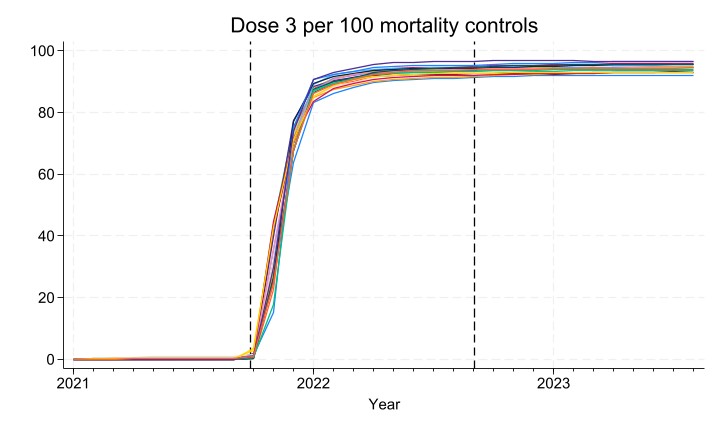

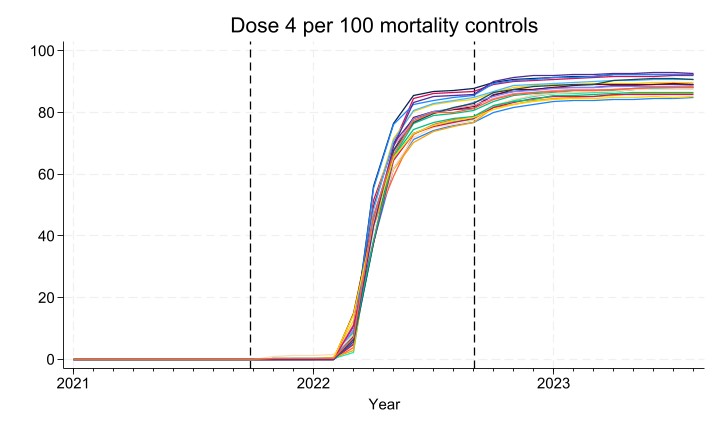

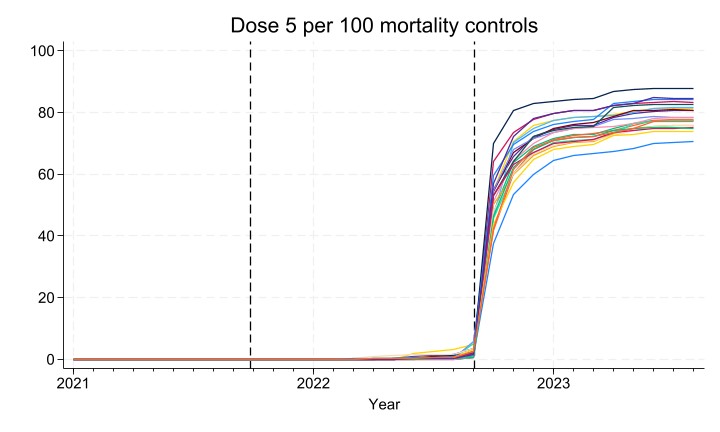

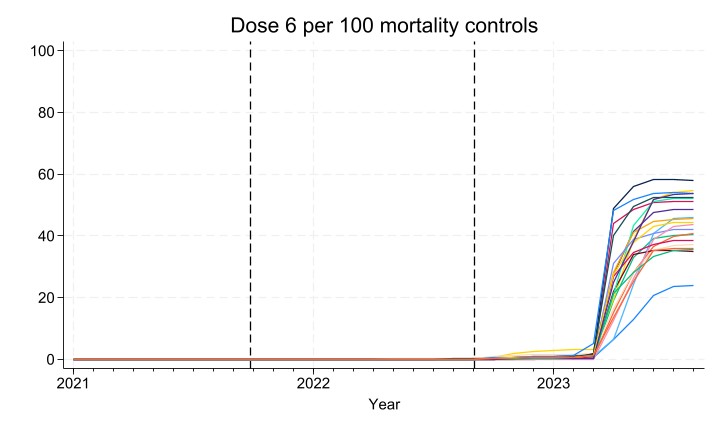

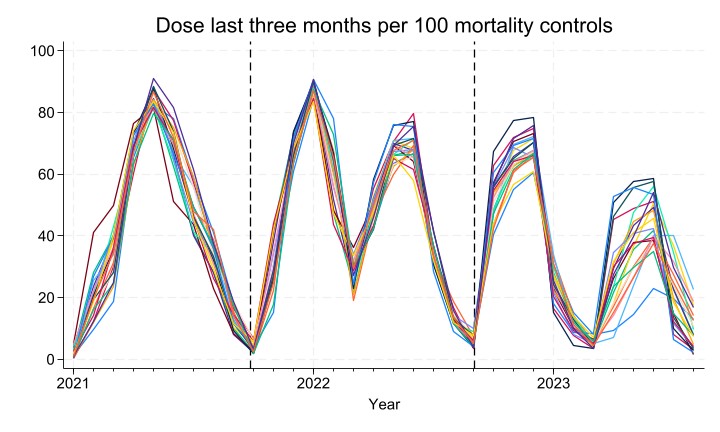

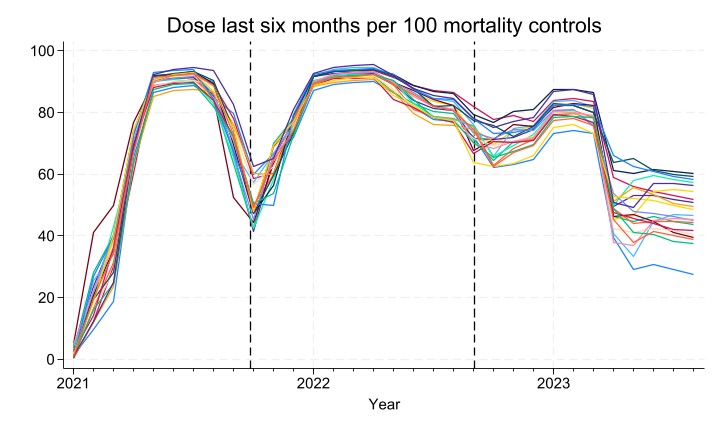


**Fig S9.** Vaccination coverage over time among the controls (whether having received doses 1–6, and whether having received at least one dose during the last three or six months). Each coloured line represents a county. The different study periods are separated by dashed lines.


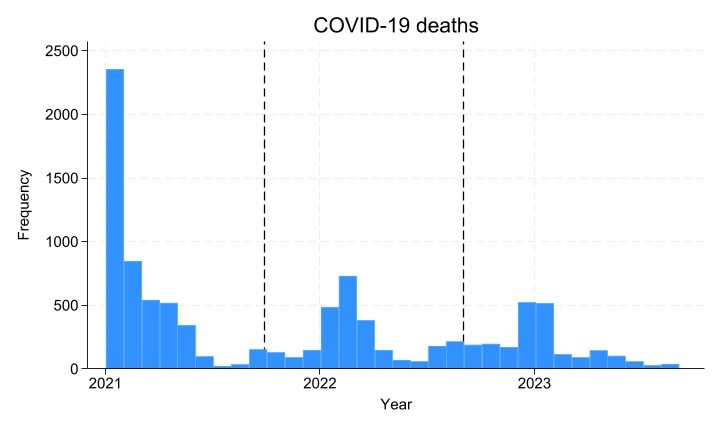


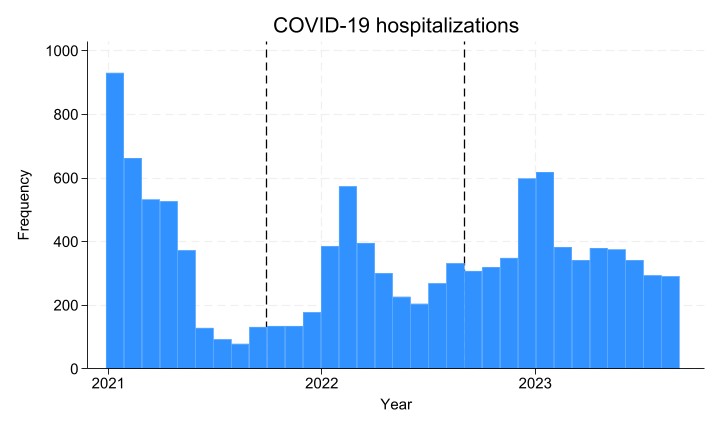


**Fig S10.** COVID-19 deaths and hospitalizations over time. The different study periods are separated by dashed lines. Data are based on the full cohort.


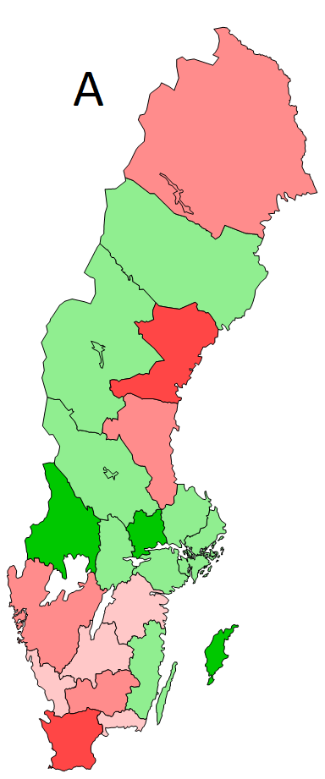

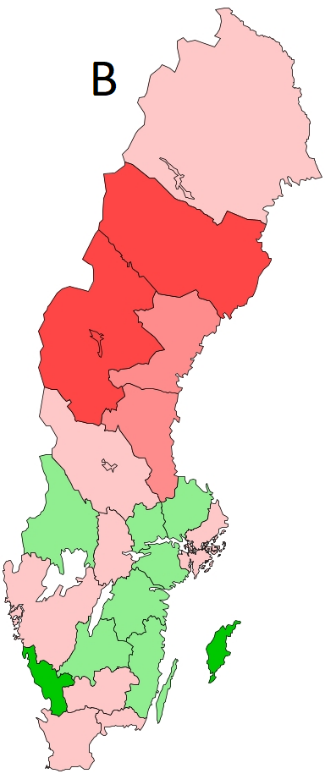

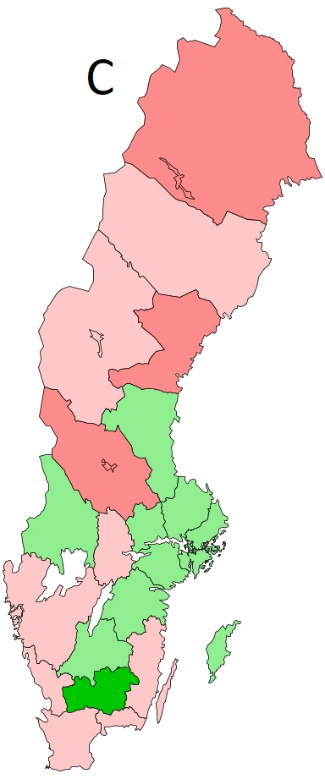


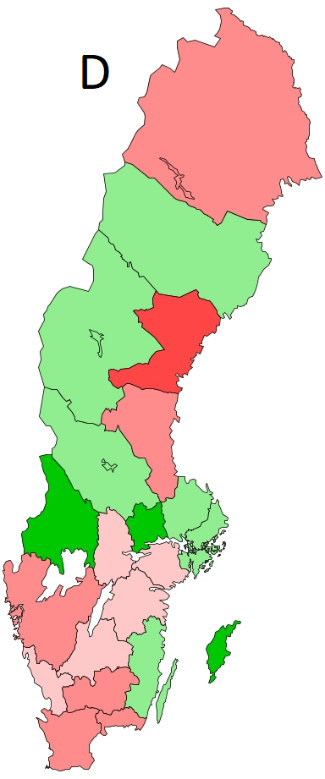

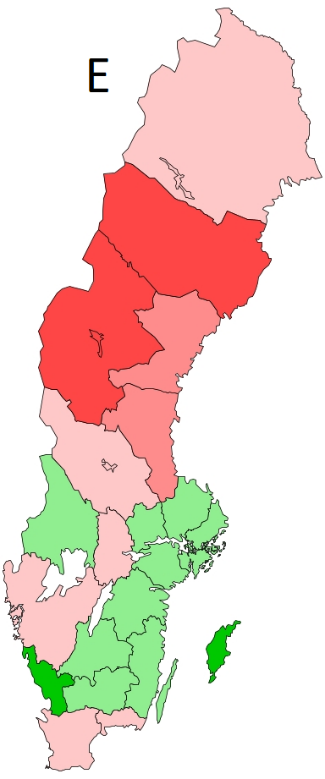

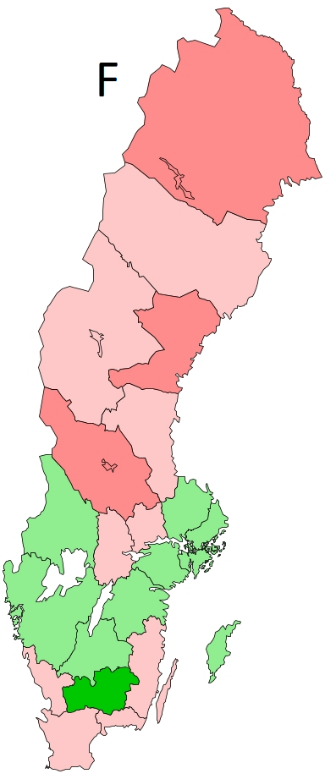


| 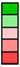 | -59% to -30% |
| --- | --- |
|  | -30% to ±0% |
|  | ±0% to +30% |
|  | +30% to +60% |
|  | +60% to +92% |

**Fig S11.** Relative COVID-19 mortality across Swedish counties. Panels A–C show relative mortality from Model 1 (no adjustment for vaccination uptake) and panels D–F show relative mortality from Model 2 (adjustment for vaccination uptake). Panels A and D represent period 1 (January 1, 2021 – September 27, 2021), panels B and E period 2 (September 28, 2021 – August 31, 2022), and panels C and F period 3 (September 1, 2022 – August 31, 2023). Mortality is expressed in relation to an “average” county.


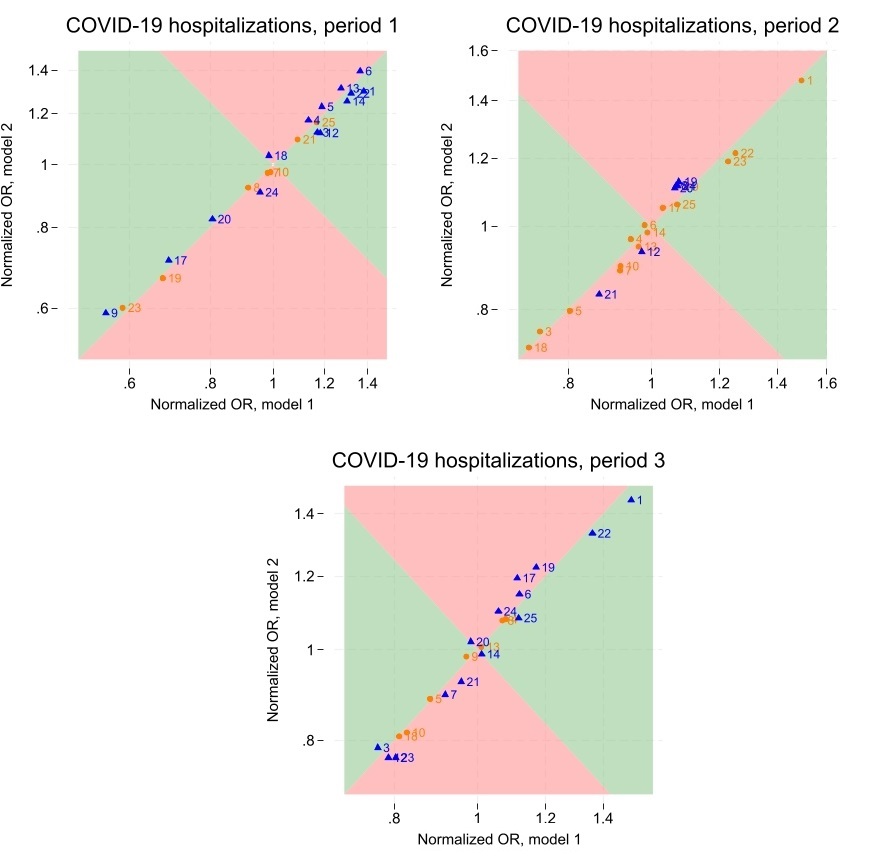


**Fig S12**. Normalized odds ratios for COVID-19 hospitalization from Models 1 and 2. The figure shows results for the three periods: Period 1, 2, and 3. Period 1 covers the rollout of doses 1 and 2 (January 1, 2021 – September 27, 2021); period 2 covers the rollout of doses 3 and 4 (September 28, 2021 – August 31, 2022); period 3 covers the rollout of doses 5 and 6 (September 1, 2022 – August 31, 2023). The blue triangles represent counties for which the normalized odds ratios from the two models were statistically different at the 0.05 level; other counties are represented by orange circles. For counties in the green areas, estimates from Model 2 were less extreme than those from Model 1; for counties in the red areas, the opposite holds. The numbers represent the official county numbers (indicated in Table 2).


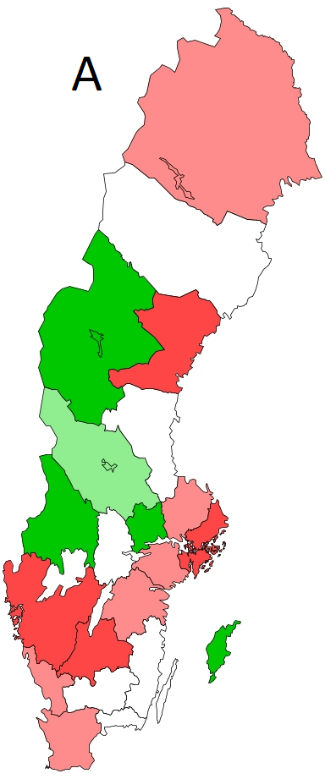

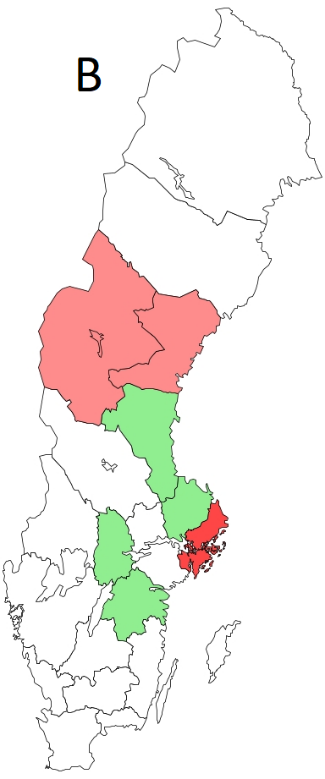

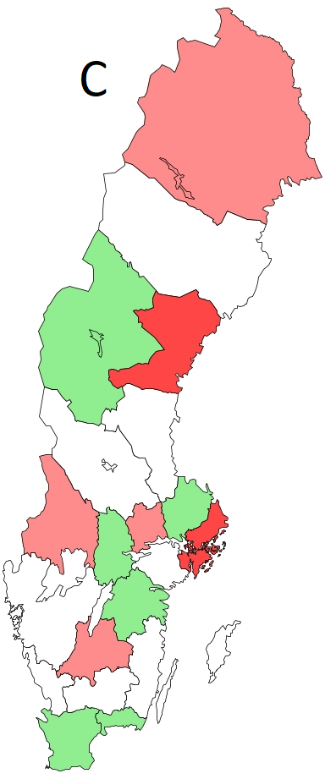


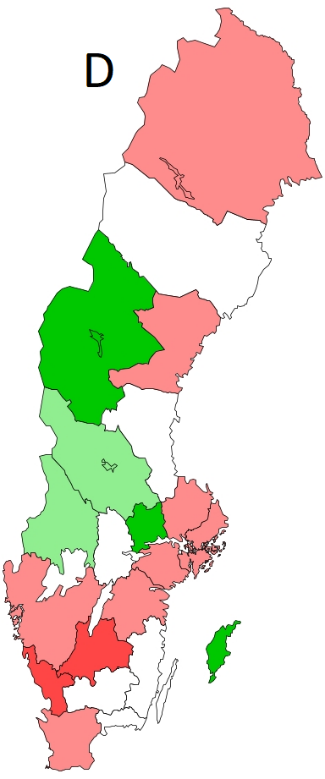

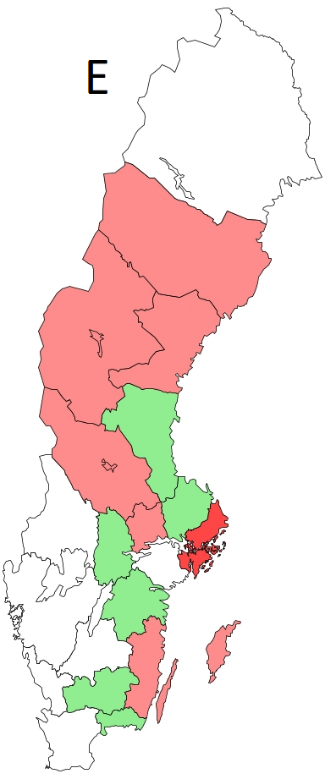

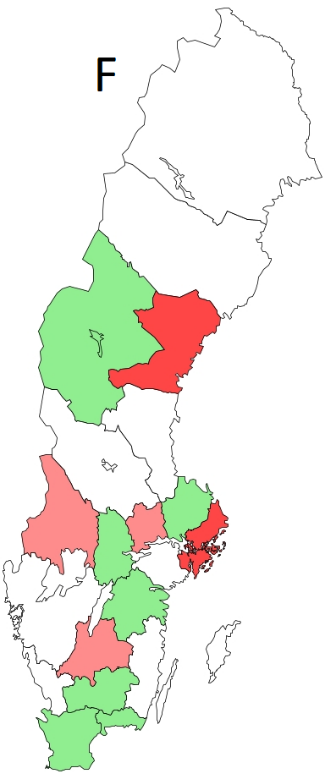


| 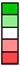 | -46% to -30% |
| --- | --- |
|  | -30% to -10% |
|  | -10% to +10% |
|  | +10% to +30% |
|  | +30% to +52% |

**Fig S13.** Relative COVID-19 hospitalizations across Swedish counties. Panels A-C show relative mortality from Model 1 (no adjustment for vaccination uptake) and panels D–F show relative mortality from Model 2 (adjustment for vaccination uptake). Panels A and D represent period 1 (January 1, 2021 – September 27, 2021), panels B and E period 2 (September 28, 2021 – August 31, 2022), and panels C and F period 3 (September 1, 2022 – August 31, 2023). Hospitalizations are expressed in relation to an “average” county.

**Table S1.** Descriptive statistics (COVID-19 mortality)

|  | Period 1 | | Period 2 | | Period 3 | |
| --- | --- | --- | --- | --- | --- | --- |
| Mortality ratios (95% CI) | Cases | Controls | Cases | Controls | Cases | Controls |
| County |  |  |  |  |  |  |
| 1 Stockholm | 848 (19.3%) | 7,817 (17.8%) | 534 (20.8%) | 4,537 (17.7%) | 359 (16.9%) | 3,796 (17.8%) |
| 3 Uppsala | 128 (2.9%) | 1,479 (3.4%) | 66 (2.6%) | 920 (3.6%) | 60 (2.8%) | 732 (3.4%) |
| 4 Södermanland | 106 (2.4%) | 1,402 (3.2%) | 75 (2.9%) | 802 (3.1%) | 68 (3.2%) | 709 (3.3%) |
| 5 Östergötland | 208 (4.7%) | 2,062 (4.7%) | 103 (4.0%) | 1,236 (4.8%) | 85 (4.0%) | 990 (4.7%) |
| 6 Jönköping | 176 (4.0%) | 1,610 (3.7%) | 79 (3.1%) | 945 (3.7%) | 82 (3.9%) | 811 (3.8%) |
| 7 Kronoberg | 101 (2.3%) | 922 (2.1%) | 55 (2.1%) | 566 (2.2%) | 31 (1.5%) | 456 (2.1%) |
| 8 Kalmar | 98 (2.2%) | 1,328 (3.0%) | 61 (2.4%) | 775 (3.0%) | 71 (3.3%) | 676 (3.2%) |
| 9 Gotland | 11 (0.3%) | 335 (0.8%) | 7 (0.3%) | 198 (0.8%) | 15 (0.7%) | 157 (0.7%) |
| 10 Blekinge | 77 (1.8%) | 841 (1.9%) | 42 (1.6%) | 483 (1.9%) | 44 (2.1%) | 424 (2.0%) |
| 12 Skåne | 778 (17.7%) | 5,767 (13.1%) | 361 (14.0%) | 3,406 (13.3%) | 268 (12.6%) | 2,691 (12.6%) |
| 13 Halland | 151 (3.4%) | 1,611 (3.7%) | 63 (2.5%) | 921 (3.6%) | 84 (3.9%) | 753 (3.5%) |
| 14 Västra Götaland | 828 (18.8%) | 7,249 (16.5%) | 417 (16.2%) | 4,177 (16.3%) | 342 (16.1%) | 3,576 (16.8%) |
| 17 Värmland | 78 (1.8%) | 1,546 (3.5%) | 68 (2.6%) | 848 (3.3%) | 72 (3.4%) | 752 (3.5%) |
| 18 Örebro | 96 (2.2%) | 1,385 (3.2%) | 72 (2.8%) | 800 (3.1%) | 66 (3.1%) | 694 (3.3%) |
| 19 Västmanland | 63 (1.4%) | 1,313 (3.0%) | 75 (2.9%) | 795 (3.1%) | 68 (3.2%) | 648 (3.0%) |
| 20 Dalarna | 93 (2.1%) | 1,532 (3.5%) | 88 (3.4%) | 905 (3.5%) | 91 (4.3%) | 721 (3.4%) |
| 21 Gävleborg | 155 (3.5%) | 1,373 (3.1%) | 97 (3.8%) | 792 (3.1%) | 75 (3.5%) | 664 (3.1%) |
| 22 Västernorrland | 152 (3.5%) | 1,193 (2.7%) | 85 (3.3%) | 730 (2.8%) | 74 (3.5%) | 541 (2.5%) |
| 23 Jämtland | 32 (0.7%) | 691 (1.6%) | 46 (1.8%) | 421 (1.6%) | 29 (1.4%) | 305 (1.4%) |
| 24 Västerbotten | 82 (1.9%) | 1,236 (2.8%) | 101 (3.9%) | 699 (2.7%) | 58 (2.7%) | 591 (2.8%) |
| 25 Norrbotten | 133 (3.0%) | 1,230 (2.8%) | 76 (3.0%) | 734 (2.9%) | 87 (4.1%) | 589 (2.8%) |
| Foreign-born | 849 (19.3%) | 5,423 (12.3%) | 470 (18.3%) | 3,137 (12.2%) | 260 (12.2%) | 2,550 (12.0%) |
| Married/registered partner | 1,632 (37.1%) | 19,357 (44.1%) | 868 (33.8%) | 10,675 (41.6%) | 776 (36.4%) | 9,085 (42.7%) |
| Nursing home resident | 1,340 (30.5%) | 4,431 (10.1%) | 1,021 (39.7%) | 3,177 (12.4%) | 872 (41.0%) | 2,672 (12.6%) |
| Receiving home assistance | 1,317 (30.0%) | 8,762 (19.9%) | 905 (35.2%) | 6,126 (23.8%) | 789 (37.1%) | 5,323 (25.0%) |
| Share secondary education | 0.45 (0.38-0.52) | 0.47 (0.38-0.54) | 0.46 (0.38-0.53) | 0.46 (0.38-0.54) | 0.47 (0.40-0.54) | 0.47 (0.38-0.54) |
| Share tertiary education | 0.38 (0.30-0.51) | 0.38 (0.30-0.52) | 0.37 (0.30-0.50) | 0.39 (0.30-0.52) | 0.37 (0.30-0.50) | 0.38 (0.30-0.52) |
| Comorbidities |  |  |  |  |  |  |
| Cardiovascular disease | 3,333 (75.9%) | 19,880 (45.3%) | 1,928 (75.0%) | 12,072 (47.0%) | 1,595 (74.9%) | 10,052 (47.2%) |
| Diabetes or obesity | 1,377 (31.3%) | 5,521 (12.6%) | 672 (26.1%) | 3,215 (12.5%) | 550 (25.8%) | 2,691 (12.6%) |
| Kidney or liver disease | 620 (14.1%) | 1,586 (3.6%) | 397 (15.4%) | 1,075 (4.2%) | 324 (15.2%) | 889 (4.2%) |
| Respiratory disease | 1,913 (43.5%) | 4,032 (9.2%) | 1,016 (39.5%) | 2,376 (9.2%) | 791 (37.2%) | 2,018 (9.5%) |
| Neurological disease | 1,680 (38.2%) | 6,632 (15.1%) | 1,052 (40.9%) | 4,109 (16.0%) | 902 (42.4%) | 3,436 (16.1%) |
| Cancer/immunosuppressed | 1,099 (25.0%) | 8,728 (19.9%) | 678 (26.4%) | 5,350 (20.8%) | 540 (25.4%) | 4,606 (21.6%) |
| Other comorbidity | 445 (10.1%) | 1,717 (3.9%) | 256 (10.0%) | 881 (3.4%) | 217 (10.2%) | 759 (3.6%) |
| Last vaccination |  |  |  |  |  |  |
| Last 3 months | 942 (21.4%) | 11,210 (25.5%) | 489 (19.0%) | 10,531 (41.0%) | 412 (19.4%) | 6,989 (32.8%) |
| 3-6 months ago | 81 (1.8%) | 1,346 (3.1%) | 1,049 (40.8%) | 11,549 (45.0%) | 720 (33.8%) | 7,963 (37.4%) |
| 6-9 months ago | 80 (1.8%) | 418 (1.0%) | 290 (11.3%) | 1,829 (7.1%) | 372 (17.5%) | 2,871 (13.5%) |
| 9-12 months ago | 0 (0.0%) | 0 (0.0%) | 161 (6.3%) | 547 (2.1%) | 172 (8.1%) | 1,266 (6.0%) |
| Further back/never | 3,291 (74.9%) | 30,948 (70.5%) | 582 (22.6%) | 1,234 (4.8%) | 453 (21.3%) | 2,187 (10.3%) |
| Number of doses |  |  |  |  |  |  |
| 0 | 3,291 (74.9%) | 30,948 (70.5%) | 543 (21.1%) | 1,070 (4.2%) | 246 (11.6%) | 760 (3.6%) |
| 1 | 746 (17.0%) | 6,623 (15.1%) | 50 (1.9%) | 139 (0.5%) | 22 (1.0%) | 58 (0.3%) |
| 2 | 354 (8.1%) | 6,312 (14.4%) | 477 (18.6%) | 3,085 (12.0%) | 73 (3.4%) | 461 (2.2%) |
| 3 | 3 (0.1%) | 37 (0.1%) | 1,060 (41.2%) | 15,861 (61.7%) | 214 (10.1%) | 1,583 (7.4%) |
| 4 | 0 (0.0%) | 2 (0.0%) | 432 (16.8%) | 5,485 (21.4%) | 499 (23.4%) | 4,364 (20.5%) |
| 5 | 0 (0.0%) | 0 (0.0%) | 9 (0.4%) | 49 (0.2%) | 989 (46.5%) | 12,641 (59.4%) |
| 6+ | 0 (0.0%) | 0 (0.0%) | 0 (0.0%) | 1 (0.0%) | 86 (4.0%) | 1,409 (6.6%) |
| N | 4,394 | 43,922 | 2,571 | 25,690 | 2,129 | 21,276 |

Notes: Descriptive statistics for the COVID-19 mortality analysis sample for the three periods. Period 1 covers the rollout of doses 1 and 2 (January 1, 2021 – September 27, 2021); period 2 covers the rollout of doses 3 and 4 (September 28, 2021 – August 31, 2022); period 3 covers the rollout of doses 5 and 6 (September 1, 2022 – August 31, 2023). For “Share secondary education” and “Share tertiary education,” numbers represent (area-specific) medians and interquartile ranges.

**Table S2.** Descriptive statistics (COVID-19 hospitalizations)

|  | Period 1 | | Period 2 | | Period 3 | |
| --- | --- | --- | --- | --- | --- | --- |
| Hospitalization ratios (95% CI) | Case | Control | Case | Control | Case | Control |
| County |  |  |  |  |  |  |
| 1 Stockholm | 3,089 (25.8%) | 24,507 (18.6%) | 3,429 (29.2%) | 24,210 (18.8%) | 2,875 (26.0%) | 22,341 (18.4%) |
| 3 Uppsala | 387 (3.2%) | 4,524 (3.4%) | 278 (2.4%) | 4,375 (3.4%) | 266 (2.4%) | 3,995 (3.3%) |
| 4 Södermanland | 365 (3.0%) | 4,245 (3.2%) | 321 (2.7%) | 4,075 (3.2%) | 356 (3.2%) | 4,033 (3.3%) |
| 5 Östergötland | 551 (4.6%) | 6,075 (4.6%) | 406 (3.5%) | 5,948 (4.6%) | 437 (3.9%) | 5,602 (4.6%) |
| 6 Jönköping | 524 (4.4%) | 4,937 (3.8%) | 424 (3.6%) | 4,784 (3.7%) | 474 (4.3%) | 4,652 (3.8%) |
| 7 Kronoberg | 194 (1.6%) | 2,651 (2.0%) | 195 (1.7%) | 2,606 (2.0%) | 208 (1.9%) | 2,634 (2.2%) |
| 8 Kalmar | 251 (2.1%) | 3,874 (2.9%) | 352 (3.0%) | 4,087 (3.2%) | 354 (3.2%) | 3,759 (3.1%) |
| 9 Gotland | 36 (0.3%) | 938 (0.7%) | 83 (0.7%) | 989 (0.8%) | 81 (0.7%) | 904 (0.7%) |
| 10 Blekinge | 165 (1.4%) | 2,381 (1.8%) | 175 (1.5%) | 2,460 (1.9%) | 169 (1.5%) | 2,323 (1.9%) |
| 12 Skåne | 1,665 (13.9%) | 17,196 (13.1%) | 1,427 (12.2%) | 16,693 (12.9%) | 1,072 (9.7%) | 15,812 (13.0%) |
| 13 Halland | 406 (3.4%) | 4,654 (3.5%) | 347 (3.0%) | 4,698 (3.6%) | 361 (3.3%) | 4,353 (3.6%) |
| 14 Västra Götaland | 2,139 (17.9%) | 21,876 (16.6%) | 1,629 (13.9%) | 20,763 (16.1%) | 1,584 (14.3%) | 19,380 (15.9%) |
| 17 Värmland | 200 (1.7%) | 4,195 (3.2%) | 336 (2.9%) | 4,295 (3.3%) | 384 (3.5%) | 4,149 (3.4%) |
| 18 Örebro | 280 (2.3%) | 3,858 (2.9%) | 227 (1.9%) | 3,886 (3.0%) | 271 (2.4%) | 3,818 (3.1%) |
| 19 Västmanland | 216 (1.8%) | 3,722 (2.8%) | 379 (3.2%) | 3,849 (3.0%) | 373 (3.4%) | 3,599 (3.0%) |
| 20 Dalarna | 247 (2.1%) | 4,381 (3.3%) | 368 (3.1%) | 4,492 (3.5%) | 355 (3.2%) | 4,254 (3.5%) |
| 21 Gävleborg | 355 (3.0%) | 4,487 (3.4%) | 302 (2.6%) | 4,164 (3.2%) | 340 (3.1%) | 4,040 (3.3%) |
| 22 Västernorrland | 321 (2.7%) | 3,798 (2.9%) | 328 (2.8%) | 3,654 (2.8%) | 366 (3.3%) | 3,424 (2.8%) |
| 23 Jämtland | 65 (0.5%) | 1,864 (1.4%) | 148 (1.3%) | 1,793 (1.4%) | 125 (1.1%) | 1,809 (1.5%) |
| 24 Västerbotten | 221 (1.8%) | 3,670 (2.8%) | 275 (2.3%) | 3,568 (2.8%) | 282 (2.5%) | 3,337 (2.7%) |
| 25 Norrbotten | 295 (2.5%) | 3,800 (2.9%) | 308 (2.6%) | 3,658 (2.8%) | 334 (3.0%) | 3,476 (2.9%) |
| Foreign-born | 3,057 (25.5%) | 18,175 (13.8%) | 2,421 (20.6%) | 16,947 (13.1%) | 1,591 (14.4%) | 15,233 (12.5%) |
| Married/registered partner | 5,548 (46.3%) | 66,713 (50.7%) | 4,954 (42.2%) | 61,622 (47.8%) | 4,774 (43.1%) | 57,873 (47.6%) |
| Nursing home resident | 751 (6.3%) | 6,731 (5.1%) | 1,012 (8.6%) | 9,601 (7.4%) | 944 (8.5%) | 9,786 (8.0%) |
| Receiving home assistance | 2,623 (21.9%) | 16,487 (12.5%) | 4,510 (38.4%) | 23,851 (18.5%) | 4,650 (42.0%) | 24,792 (20.4%) |
| Share secondary education | 0.44 (0.37-0.52) | 0.47 (0.38-0.54) | 0.44 (0.37-0.52) | 0.47 (0.38-0.54) | 0.46 (0.38-0.53) | 0.47 (0.38-0.54) |
| Share tertiary education | 0.39 (0.30-0.52) | 0.38 (0.30-0.52) | 0.39 (0.30-0.52) | 0.38 (0.30-0.52) | 0.38 (0.30-0.51) | 0.38 (0.30-0.52) |
| Comorbidities |  |  |  |  |  |  |
| Cardiovascular disease | 5,120 (42.8%) | 34,575 (26.3%) | 5,328 (45.4%) | 36,749 (28.5%) | 4,876 (44.1%) | 34,977 (28.7%) |
| Diabetes or obesity | 2,461 (20.6%) | 12,498 (9.5%) | 2,254 (19.2%) | 12,472 (9.7%) | 1,878 (17.0%) | 11,554 (9.5%) |
| Kidney or liver disease | 756 (6.3%) | 2,742 (2.1%) | 877 (7.5%) | 3,187 (2.5%) | 653 (5.9%) | 2,749 (2.3%) |
| Respiratory disease | 2,318 (19.4%) | 8,524 (6.5%) | 2,239 (19.1%) | 8,560 (6.6%) | 1,979 (17.9%) | 7,948 (6.5%) |
| Neurological disease | 2,057 (17.2%) | 13,081 (9.9%) | 2,455 (20.9%) | 13,939 (10.8%) | 2,336 (21.1%) | 13,206 (10.9%) |
| Cancer/immunosuppressed | 2,536 (21.2%) | 23,348 (17.7%) | 3,143 (26.8%) | 25,880 (20.1%) | 2,905 (26.2%) | 25,067 (20.6%) |
| Other comorbidity | 752 (6.3%) | 4,388 (3.3%) | 776 (6.6%) | 3,881 (3.0%) | 629 (5.7%) | 3,444 (2.8%) |
| Last vaccination |  |  |  |  |  |  |
| Last 3 months | 2,286 (19.1%) | 32,976 (25.1%) | 2,861 (24.4%) | 55,406 (42.9%) | 2,566 (23.2%) | 41,740 (34.3%) |
| 3-6 months ago | 211 (1.8%) | 3,549 (2.7%) | 4,627 (39.4%) | 53,675 (41.6%) | 2,980 (26.9%) | 37,120 (30.5%) |
| 6-9 months ago | 101 (0.8%) | 704 (0.5%) | 1,428 (12.2%) | 9,715 (7.5%) | 2,295 (20.7%) | 20,864 (17.1%) |
| 9-12 months ago | 0 (0.0%) | 0 (0.0%) | 602 (5.1%) | 2,671 (2.1%) | 1,018 (9.2%) | 8,176 (6.7%) |
| Further back/never | 9,374 (78.3%) | 94,404 (71.7%) | 2,219 (18.9%) | 7,580 (5.9%) | 2,208 (20.0%) | 13,794 (11.3%) |
| Number of doses |  |  |  |  |  |  |
| 0 | 9,374 (78.3%) | 94,404 (71.7%) | 2,036 (17.3%) | 6,690 (5.2%) | 953 (8.6%) | 4,893 (4.0%) |
| 1 | 1,882 (15.7%) | 20,964 (15.9%) | 183 (1.6%) | 715 (0.6%) | 64 (0.6%) | 377 (0.3%) |
| 2 | 715 (6.0%) | 16,184 (12.3%) | 2,111 (18.0%) | 14,674 (11.4%) | 492 (4.4%) | 3,082 (2.5%) |
| 3 | 1 (0.0%) | 77 (0.1%) | 4,968 (42.3%) | 74,323 (57.6%) | 1,341 (12.1%) | 10,446 (8.6%) |
| 4 | 0 (0.0%) | 3 (0.0%) | 2,383 (20.3%) | 32,351 (25.1%) | 2,666 (24.1%) | 26,557 (21.8%) |
| 5 | 0 (0.0%) | 1 (0.0%) | 54 (0.5%) | 286 (0.2%) | 5,045 (45.6%) | 67,899 (55.8%) |
| 6+ | 0 (0.0%) | 0 (0.0%) | 2 (0.0%) | 8 (0.0%) | 506 (4.6%) | 8,440 (6.9%) |
| N | 11,972 | 119,661 | 11,737 | 117,310 | 11,067 | 110,627 |

Notes: The table shows descriptive statistics for the COVID-19 hospitalization analysis sample. Period 1 covers the rollout of doses 1 and 2 (January 1, 2021 – September 27, 2021); period 2 covers the rollout of doses 3 and 4 (September 28, 2021 – August 31, 2022); period 3 covers the rollout of doses 5 and 6 (September 1, 2022 – August 31, 2023). For “Share secondary education” and “Share tertiary education,” numbers represent (area-specific) medians and interquartile ranges.

**Table S3.** COVID-19 hospitalization ratios

|  | Period 1 | | Period 2 | | Period 3 | |
| --- | --- | --- | --- | --- | --- | --- |
| Hospitalization ratios (95% CI) | Model 1 | Model 2 | Model 1 | Model 2 | Model 1 | Model 2 |
| Foreign-born | 1.95 (1.85-2.05) | 1.83 (1.74-1.92) | 1.53 (1.45-1.62) | 1.16 (1.09-1.22) | 1.10 (1.04-1.17) | 0.92 (0.86-0.98) |
| Married/registered partner | 0.93 (0.89-0.97) | 0.95 (0.91-0.99) | 0.94 (0.90-0.99) | 1.07 (1.03-1.12) | 0.97 (0.93-1.02) | 1.05 (1.00-1.10) |
| Nursing home resident | 1.50 (1.37-1.65) | 2.83 (2.56-3.14) | 1.89 (1.74-2.05) | 1.86 (1.71-2.03) | 1.76 (1.62-1.91) | 1.88 (1.73-2.05) |
| Receiving home assistance | 1.93 (1.82-2.05) | 2.39 (2.25-2.54) | 3.35 (3.19-3.53) | 3.23 (3.06-3.41) | 3.56 (3.38-3.74) | 3.45 (3.28-3.63) |
| Share secondary education | 0.04 (0.02-0.06) | 0.04 (0.02-0.06) | 0.05 (0.03-0.08) | 0.13 (0.08-0.22) | 0.69 (0.42-1.13) | 1.18 (0.72-1.94) |
| Share tertiary education | 0.09 (0.07-0.13) | 0.10 (0.07-0.13) | 0.11 (0.08-0.15) | 0.24 (0.17-0.34) | 0.48 (0.33-0.68) | 0.79 (0.55-1.13) |
| Comorbidities |  |  |  |  |  |  |
| Cardiovascular disease | 1.49 (1.43-1.56) | 1.51 (1.44-1.58) | 1.40 (1.34-1.47) | 1.45 (1.39-1.52) | 1.38 (1.31-1.44) | 1.39 (1.33-1.46) |
| Diabetes or obesity | 1.71 (1.62-1.81) | 1.76 (1.66-1.86) | 1.47 (1.38-1.55) | 1.48 (1.39-1.57) | 1.34 (1.26-1.43) | 1.37 (1.28-1.45) |
| Kidney or liver disease | 1.92 (1.74-2.11) | 1.98 (1.79-2.18) | 2.01 (1.83-2.20) | 2.04 (1.86-2.25) | 1.75 (1.58-1.93) | 1.78 (1.60-1.97) |
| Respiratory disease | 3.02 (2.86-3.20) | 3.07 (2.90-3.26) | 2.79 (2.63-2.95) | 2.80 (2.64-2.98) | 2.66 (2.51-2.83) | 2.68 (2.52-2.85) |
| Neurological disease | 1.43 (1.35-1.51) | 1.48 (1.39-1.57) | 1.62 (1.54-1.72) | 1.70 (1.61-1.80) | 1.70 (1.61-1.80) | 1.76 (1.66-1.86) |
| Cancer/immunosuppressed | 1.20 (1.15-1.27) | 1.23 (1.17-1.29) | 1.42 (1.35-1.48) | 1.53 (1.46-1.61) | 1.34 (1.27-1.40) | 1.39 (1.32-1.46) |
| Other comorbidity | 1.33 (1.22-1.45) | 1.34 (1.22-1.47) | 1.42 (1.29-1.55) | 1.30 (1.19-1.43) | 1.31 (1.19-1.45) | 1.24 (1.12-1.37) |
| Last vaccination |  |  |  |  |  |  |
| Last 3 months |  | 0.52 (0.38-0.73) |  | 0.28 (0.22-0.35) |  | 0.45 (0.39-0.52) |
| 3-6 months ago |  | 0.53 (0.39-0.71) |  | 0.53 (0.42-0.65) |  | 0.66 (0.58-0.75) |
| 6-9 months ago |  |  |  | 0.85 (0.70-1.05) |  | 0.90 (0.80-1.02) |
| 9-12 months ago |  |  |  | 0.95 (0.77-1.17) |  | 0.96 (0.86-1.07) |
| Further back/never (ref.) |  | 1.00 |  | 1.00 |  | 1.00 |
| Number of doses |  |  |  |  |  |  |
| 0 (ref.) |  | 1.00 |  | 1.00 |  | 1.00 |
| 1 |  | 1.10 (0.79-1.54) |  | 0.89 (0.68-1.16) |  | 0.77 (0.57-1.04) |
| 2 |  | 0.20 (0.14-0.27) |  | 0.53 (0.44-0.65) |  | 0.68 (0.60-0.77) |
| 3 |  |  |  | 0.35 (0.28-0.44) |  | 0.55 (0.49-0.61) |
| 4 |  |  |  | 0.48 (0.37-0.63) |  | 0.50 (0.44-0.58) |
| 5 |  |  |  |  |  | 0.49 (0.42-0.57) |
| 6 |  |  |  |  |  | 0.51 (0.42-0.63) |
| N | 131,633 | 131,633 | 129,047 | 129,047 | 121,694 | 121,694 |

Notes: COVID-19 hospitalization ratios from Models 1 and 2, estimated with logistic regression on the case-control dataset on hospitalizations. The table shows results for the full time period (January 2021 – August 2023) as well as the three subperiods: Period 1, 2, and 3. Period 1 covers the rollout of doses 1 and 2 (January 1, 2021 – September 27, 2021); period 2 covers the rollout of doses 3 and 4 (September 28, 2021 – August 31, 2022); period 3 covers the rollout of doses 5 and 6 (September 1, 2022 – August 31, 2023). Results for county effects are reported as comparisons with an “average” county (the geometric average of the county-specific mortality ratios is equal to 1). Effect estimates for the highest category of “Number of doses” should be interpreted at “at least this number of doses” (at least 6 doses for the full period and period 3, at least 2 doses for period 1, and at least 4 doses for period 2). 95% confidence intervals are given within parentheses. All models were additionally adjusted for county fixed effects (shown in Figure 3).

# **Table S4.** COVID-19 hospitalizations, PIF, and AHR

|  | Period 1 | | | Period 2 | | | Period 3 | | |
| --- | --- | --- | --- | --- | --- | --- | --- | --- | --- |
| County | Actual hospitalizations | PIF | AHR | Actual hospitalizations | PIF | AHR | Actual hospitalizations | PIF | AHR |
| 1 Stockholm | 3115 | 0.09 (0.08-0.09) | 72 (68-76) | 3407 | 0.12 (0.11-0.12) | 109 (104-115) | 2877 | 0.13 (0.12-0.14) | 107 (100-115) |
| 3 Uppsala | 387 | 0.09 (0.08-0.09) | 47 (44-50) | 280 | 0.02 (0.02-0.02) | 10 (9-10) | 266 | 0.02 (0.02-0.02) | 9 (8-9) |
| 4 Södermanland | 365 | 0.03 (0.03-0.04) | 19 (18-20) | 321 | 0.07 (0.06-0.07) | 33 (31-35) | 353 | 0.09 (0.08-0.09) | 51 (48-55) |
| 5 Östergötland | 553 | 0.03 (0.03-0.03) | 18 (17-20) | 405 | 0.03 (0.03-0.04) | 15 (14-16) | 438 | 0.05 (0.04-0.05) | 23 (22-25) |
| 6 Jönköping | 525 | 0.03 (0.03-0.03) | 23 (22-25) | 421 | 0.04 (0.04-0.04) | 25 (24-26) | 476 | 0.06 (0.05-0.06) | 41 (38-44) |
| 7 Kronoberg | 194 | 0.07 (0.07-0.08) | 34 (32-36) | 195 | 0.09 (0.09-0.10) | 44 (42-46) | 212 | 0.08 (0.07-0.09) | 44 (41-47) |
| 8 Kalmar | 251 | 0.04 (0.03-0.04) | 18 (17-19) | 348 | 0.03 (0.03-0.03) | 19 (18-20) | 356 | 0.08 (0.07-0.08) | 49 (46-52) |
| 9 Gotland | 36 | 0.01 (0.01-0.01) | 2 (2-2) | 83 | 0.05 (0.05-0.05) | 29 (28-30) | 78 | 0.07 (0.07-0.08) | 39 (37-42) |
| 10 Blekinge | 166 | 0.04 (0.03-0.04) | 16 (16-17) | 174 | 0.06 (0.06-0.06) | 30 (29-32) | 167 | 0.07 (0.07-0.08) | 35 (33-38) |
| 12 Skåne | 1670 | 0.07 (0.07-0.08) | 45 (42-48) | 1426 | 0.10 (0.09-0.10) | 55 (53-58) | 1078 | 0.07 (0.07-0.08) | 33 (31-35) |
| 13 Halland | 406 | 0.05 (0.05-0.06) | 30 (28-32) | 351 | 0.06 (0.06-0.07) | 32 (30-34) | 355 | 0.06 (0.05-0.06) | 30 (28-32) |
| 14 Västra Götaland | 2144 | 0.07 (0.07-0.08) | 47 (45-50) | 1632 | 0.09 (0.09-0.09) | 46 (44-49) | 1587 | 0.08 (0.08-0.09) | 42 (40-45) |
| 17 Värmland | 201 | 0.03 (0.03-0.03) | 8 (8-9) | 339 | 0.01 (0.01-0.01) | 7 (7-8) | 382 | 0.01 (0.01-0.01) | 5 (5-5) |
| 18 Örebro | 281 | 0.03 (0.03-0.03) | 13 (12-14) | 229 | 0.05 (0.05-0.05) | 18 (17-19) | 275 | 0.09 (0.08-0.10) | 41 (39-44) |
| 19 Västmanland | 222 | 0.05 (0.05-0.06) | 20 (19-22) | 373 | 0.06 (0.06-0.06) | 41 (39-43) | 370 | 0.04 (0.04-0.04) | 27 (25-29) |
| 20 Dalarna | 247 | 0.04 (0.04-0.04) | 14 (13-14) | 368 | 0.03 (0.03-0.03) | 17 (16-18) | 354 | 0.03 (0.03-0.03) | 17 (16-18) |
| 21 Gävleborg | 355 | 0.05 (0.05-0.05) | 27 (25-28) | 304 | 0.06 (0.06-0.07) | 30 (29-32) | 341 | 0.07 (0.06-0.07) | 38 (35-40) |
| 22 Västernorrland | 321 | 0.06 (0.06-0.07) | 36 (33-38) | 328 | 0.07 (0.07-0.07) | 41 (39-42) | 366 | 0.09 (0.08-0.09) | 61 (57-65) |
| 23 Jämtland | 65 | 0.03 (0.03-0.03) | 6 (6-6) | 147 | 0.08 (0.07-0.08) | 38 (37-40) | 123 | 0.08 (0.07-0.08) | 35 (32-37) |
| 24 Västerbotten | 223 | 0.11 (0.11-0.12) | 44 (42-46) | 273 | 0.01 (0.01-0.01) | 3 (3-3) | 282 | 0.03 (0.03-0.04) | 18 (17-20) |
| 25 Norrbotten | 296 | 0.07 (0.07-0.08) | 37 (35-39) | 305 | 0.05 (0.05-0.05) | 27 (26-29) | 333 | 0.09 (0.08-0.09) | 53 (49-56) |
| Overall | 12,023 | 0.07 (0.06-0.07) | 40 (37-42) | 11,709 | 0.08 (0.08-0.08) | 47 (45-50) | 11,069 | 0.08 (0.08-0.09) | 48 (45-52) |

Notes: Actual COVID-19 hospitalizations, Potential Impact Fractions (PIF), and Attributable Hospitalization Rates per 100,000 individuals (AHR) for COVID-19 hospitalizations. Normal-based 95% bootstrap confidence intervals are given within parentheses. Period 1 covers the rollout of doses 1 and 2 (January 1, 2021 – September 27, 2021); period 2 covers the rollout of doses 3 and 4 (September 28, 2021 – August 31, 2022); period 3 covers the rollout of doses 5 and 6 (September 1, 2022 – August 31, 2023).
